# Identification of novel pyrazolo[4,3-c]pyridine and diazepane derivatives as potent inhibitors of *Mycobacterium tuberculosis* protein tyrosine phosphatase B

**DOI:** 10.1128/iai.00738-25

**Published:** 2026-02-09

**Authors:** Raunak Raunak, Aayush Bahl, Shubham Srivastava, Roopshali Rakshit, Shivani Bansal, Sashi Kant, Chandi C. Mandal, Saurabh Pandey, Deeksha Tripathi

**Affiliations:** 1 Microbial Pathogenesis and Microbiome Lab, Department of Microbiology, School of Life Sciences, Central University of Rajasthan 206414, Ajmer, Rajasthan, India; 2 Department of Pharmacy, School of Chemical Sciences and Pharmacy, Central University of Rajasthan 206414, Ajmer, Rajasthan, India; 3 Department of Biochemistry, School of Life Sciences, Central University of Rajasthan 726899, Ajmer, Rajasthan, India; 4 Department of Immunology and Microbiology, University of Colorado School of Medicine, Anschutz Medical Campus 12225, Aurora, Colorado, USA; 5 Department of Biochemistry, School of Chemical and Life Sciences, Jamia Hamdard 683434 , New Delhi, Delhi, India; St Jude Children's Research Hospital, Memphis, Tennessee, USA

**Keywords:** *Mycobacterium tuberculosis*, protein tyrosine phosphatase B, inhibitors, pyrazolo[4,3-c] pyridine and diazepane, drug discovery, molecular dynamics simulation, therapeutic

## Abstract

Tuberculosis (TB), caused by *Mycobacterium tuberculosis* (*M.tb*), continues to pose a critical global health threat as a leading infectious cause of mortality. Therapeutic efficacy is increasingly compromised by the emergence of multidrug-resistant strains and the limitations of existing regimens, which necessitate treatment durations of six months or longer. Protein tyrosine phosphatase B from *Mtb* (PtpB-*Mtb*) has been recognized as a critical virulence factor, representing a promising target for novel antitubercular therapies due to its unique structural and functional properties. In this study, a comprehensive structure-based virtual screening approach was employed to identify novel small-molecule scaffolds with inhibitory potential against PtpB-*Mtb*. The ChemBridge compound library was curated and filtered for drug-like properties, followed by hierarchical molecular docking and molecular dynamics simulations to prioritize candidates with high predicted affinity and stability within the PtpB-*Mtb* active site. Quantum mechanical calculations further characterized the electronic properties of top hits. Recombinant PtpB-*Mtb* was expressed and purified to homogeneity, and *in vitro* enzymatic assays were performed to evaluate the inhibitory potency and selectivity of shortlisted compounds. Two derivatives bearing pyrazolo[4,3-c]pyridine and 1,4-diazepane ring nuclei demonstrated significant inhibition of PtpB-*Mtb* activity, exhibiting IC₅₀ values of 14.4 µM and 32.6 µM, respectively. Biolayer interferometry confirmed strong and specific binding to PtpB-Mtb, with dissociation constants (K_d_) of 0.012 µM and 0.57 µM. The integrated workflow presented herein highlights the potential of these novel scaffolds as starting points for the development of selective, cell-permeable PtpB-*Mtb* inhibitors, offering a promising avenue for next-generation anti-tubercular drug discovery.

## INTRODUCTION

Tuberculosis (TB), caused by *Mycobacterium tuberculosis* (*Mtb*), remains a formidable challenge to global public health and socioeconomic stability, claiming more lives annually than any other infectious disease, as recognized by the World Health Organization (WHO) (1, 2). The global burden is further exacerbated by the prevalence of latent TB infection, which is estimated to affect up to a quarter of the world’s population, posing a continuous risk of reactivation, particularly among immune-compromised individuals (3). Recent setbacks in TB control efforts, notably due to the COVID-19 pandemic, have contributed to a resurgence in TB incidence (4). Protracted treatment regimens, escalating antimicrobial resistance, and the persistent threat of latent infection constitute the three major challenges in effective TB management, contributing to high rates of non-adherence, relapse, and the emergence of extensively drug-resistant and totally drug-resistant TB strains (1, 5). Collectively, these challenges underscore the urgent need for innovative therapeutic strategies, including the identification of novel drug targets and chemical scaffolds that can circumvent existing limitations and advance global TB control efforts.

Protein tyrosine phosphatase B of *Mtb* (PtpB-*Mtb*) has emerged as a promising target for novel anti-tubercular therapies (2, 5–11). This secreted phosphatase plays a pivotal role in the survival and replication of *Mtb* within host macrophages (12–14). Structurally characterized by a canonical phosphatase fold and a conserved active site, PtpB-*Mtb* is phylogenetically distinct from human PTPs, sharing less than 6% sequence similarity with its closest human homolog, PTP1B, allowing selective inhibition with minimal off-target effects (15, 16). Functionally, PtpB-*Mtb* acts as an essential virulence factor by subverting host immune responses and promoting intracellular persistence (16–18). PtpB-*Mtb* has been found to collectively suppress apoptosis and facilitate bacterial survival by inhibiting ERK1/2- and p38-mediated pro-inflammatory cytokine production and activating the Akt pathway (10). Exhibiting triple-specificity phosphatase activity, PtpB-*Mtb* targets phosphotyrosine, phosphoserine/threonine, and phosphoinositides like PI3P, thereby impairing phagosome maturation and host clearance (19, 20). Additionally, genetic inactivation of *PtpB-Mtb* has been found to significantly attenuate *Mtb* virulence in macrophage and animal models, further validating its role as a druggable target (21). Moreover, as PtpB-*Mtb* is secreted into the host cytosol, inhibitors can act intracellularly without the need to traverse the mycobacterial cell wall, simplifying drug delivery and enhancing therapeutic potential.

Despite advances in the identification of PtpB-Mtb inhibitors, several challenges have limited their clinical translation. Achieving selectivity remains a major obstacle, as the highly conserved and positively charged PTP active site, particularly the phosphotyrosine binding pocket, complicates the design of inhibitors that discriminate effectively between PtpB-*Mtb* and the extensive panel of human PTPs (11, 14). Early inhibitors, including natural product derivatives, often exhibited only moderate potency and poor selectivity, raising concerns regarding potential off-target effects (22–25). Cell permeability represents another major limitation (26, 27). Many potent inhibitors rely on anionic phosphomimetic groups to engage the active site, but these features frequently result in poor membrane penetration and suboptimal intracellular bioavailability. High molecular weight and overall molecular complexity further diminish drug-like properties and hinder cellular uptake. Consequently, numerous compounds that demonstrate robust *in vitro* activity fail to exhibit efficacy in cellular or animal infection models (11, 28). Moreover, translational gaps persist between *in vitro* potency and *in vivo* efficacy, frequently due to inadequate pharmacokinetic properties or rapid metabolic clearance (5, 10, 11, 29–31). The lack of detailed structural information for many inhibitor-PtpB-*Mtb* complexes further impedes rational optimization. While recent advances such as fragment-based screening and dual-site-binding strategies have improved selectivity and reduced anionic character (1), no clinically validated candidates have emerged. Thus, the development of novel scaffolds that combine high affinity, selectivity, cell permeability, and favorable pharmacological profiles remains a critical goal in the search for next-generation PtpB-*Mtb* inhibitors.

The present study was undertaken to identify and characterize novel, selective inhibitors of PtpB-*Mtb*. A structure-based virtual screening approach was employed, utilizing the X-ray crystal structure of PtpB-*Mtb* in complex with a known inhibitor to guide the identification of promising candidates. A commercially available compound library (ChemBridge) was curated and filtered for drug-like properties, followed by molecular docking to prioritize compounds with high predicted affinity for the PtpB-*Mtb* active site. Top-ranking candidates were further evaluated through molecular dynamics (MD) simulations to assess the stability of their interactions within the PtpB-Mtb-binding pocket. The most promising compounds D6 (53747349) and D9 (24807456) were subsequently subjected to *in vitro* enzymatic assays to determine their inhibitory potency and selectivity.

In contrast to many previously reported phosphatase inhibitors that suffer from poor selectivity, limited cell permeability, and suboptimal pharmacokinetic features, the present study integrates structure-guided virtual screening to identify non-anionic, heterocyclic scaffolds with balanced physicochemical properties. The identified *pyrazolo[4,3-c]pyridine* and *1,4-diazepane* derivatives exhibit low-micromolar inhibitory potency, strong binding affinity, and minimal cytotoxicity, collectively addressing key limitations of earlier PtpB-Mtb inhibitors. This approach provides a rational framework for discovering next-generation, cell-permeable inhibitors that combine selectivity, potency, and favorable pharmacological profiles.

## MATERIALS AND METHODS

### Schrödinger software and computational platform

All computational studies were conducted using Schrödinger software release 2024-1, executed on a Dell workstation equipped with Ubuntu 22.04.5 LTS, an Intel Xeon E5 chipset, and GPU acceleration via an NVIDIA P2000 5GB graphics card. Various integrated modules within the suite, including Protein Preparation Wizard (PPW), Glide, Desmond, and Jaguar, were instrumental in facilitating protein preparation, molecular docking, and MD simulations (32).

### Protein preparation and active site definition

The X-ray crystal structure of *Mtb* PtpB in complex with oxylamino-methylene-thiophene sulfonamide (OMTS) was retrieved from the Protein Data Bank (PDB ID: 2OZ5). Protein preparation was carried out using the PPW within the Schrödinger suite. The PPW protocol comprised three sequential steps: Pre-processing, Optimization, and Minimization. During pre-processing, all hydrogen atoms were added, and bond orders were corrected to ensure accurate protonation states. Missing loop regions were completed, and alternate conformations were removed. Water molecules were deleted unless they participated in key protein–ligand interactions. In the optimization step, hydrogen bonds were systematically assigned, fixed, and redefined to reflect appropriate geometries. The total energy of the protein structure was then calculated, and the conformation was refined to its lowest energy state using the OPLS4 force field in the minimization step (33). The active site of PtpB was precisely defined around the co-crystallized ligand (OMTS) using the receptor grid generation module of Glide, thereby ensuring accurate docking site localization. The final protein structure, prepared and minimized, was deemed suitable for subsequent molecular docking and dynamics studies.

### Ligand preparation and virtual screening

The commercially available ChemBridge compound library, comprising approximately 700,000 molecules, was retrieved in Structure Data File (SDF) format and utilized as the initial data set for ligand preparation. LigPrep (Schrödinger) was employed to convert the 2D structures into energetically minimized 3D conformations, ensuring accurate protonation states at neutral pH 7.0 using the Ionizer module. Stereoisomers and tautomers were generated, and the processed structures were subsequently saved in Maestro (.mae) format for compatibility with downstream workflows. The resulting library was rigorously filtered using Lipinski’s rule of five, reactive functional group exclusion, and PAINS filters to enhance drug-likeness and reduce the likelihood of false positives (34). Hierarchical molecular docking was performed using Glide (Schrödinger), employing a three-tiered screening approach that included high-throughput virtual screening, standard precision (SP), and extra precision (XP) docking modes (35). At each stage, only the top 5% of compounds based on docking scores were advanced to the next level to ensure enrichment of potential high-affinity binders. In the final XP mode, known for its superior accuracy in predicting binding poses and affinity, the top eight molecules were selected based on a combination of docking scores and knowledge-based assessment of molecular interactions. Binding free energies (ΔG) for the final compounds were estimated using the Prime MMGBSA method, with flexible residues defined within 5 Å of the ligand-binding site, enabling a hybrid implicit solvent and molecular mechanics approach to refine protein–ligand interactions (36).

To further validate the screening protocol’s ability to discriminate active compounds from inactive molecules, retrospective enrichment analysis was performed. A validation data set comprising experimentally confirmed PtpB-Mtb inhibitors compiled from literature and property-matched decoy compounds was subjected to the established Glide XP docking protocol. Receiver operating characteristic (ROC) curves were constructed by plotting true-positive rates against false-positive rates across varying docking score thresholds. The area under the ROC curve (AUC) and enrichment factors at early recognition percentages were calculated to assess the protocol’s discriminative power and early enrichment capability.

### Assessment of molecular descriptors

To further evaluate the drug-likeness of identified hits, molecular descriptors assessing absorption, distribution, metabolism, and excretion were calculated using the QikProp module in Schrodinger. Key physicochemical properties of the top eight compounds identified through virtual screening are presented, including molecular weight, hydrogen bond donors and acceptors, lipophilicity (cLogP), aqueous solubility (LogSw), total polar surface area (TPSA), and number of rotatable bonds. These descriptors were evaluated in comparison to optimal ranges for drug-like properties (37).

### MD simulations

MD simulations were performed using Desmond to assess the stability and atomistic interactions of protein–ligand complexes (38, 39). Each system was solvated in an orthorhombic SPC water box with a 10 Å buffer distance from the protein surface, neutralized with 0.15 M NaCl, and minimized using a combination of steepest descent and LBGFS algorithms. Equilibration was achieved under NPT ensemble conditions (300 K, 1 bar) using the Nose-Hoover chain thermostat and Martyna-Tobias-Klein barostat, followed by a 500 ns production run. Coulombic interactions were handled with Ewald summation, employing a 9.0 Å short-range cutoff and a 1 × 10⁻⁹ tolerance for long-range interactions. Simulation stability was assessed through several parameters, including the convergence of total potential energy and the stabilization of backbone and ligand heavy-atom RMSD values. Additional analyses, such as root mean square fluctuation (RMSF), radius of gyration (Rg), and hydrogen-bond occupancy, were performed to evaluate structural flexibility and binding stability over time. The equilibrium phase was defined based on the RMSD plateau and energy convergence profiles. MD simulation of apo PtpB-Mtb was performed under identical parameters to establish baseline protein dynamics in the absence of ligand for comparative analysis. To visualize conformational stability and binding mode persistence, representative snapshots of protein–ligand complexes were extracted at 0, 100, 200, 300, 400, and 500 ns timepoints from the MD trajectories. Snapshots were rendered in Maestro with consistent viewing angles to enable direct comparison of structural changes across the simulation timeline.

### Quantum mechanical calculations

Quantum mechanical (QM) calculations were performed to investigate the electronic properties of the top-ranked ligands identified from the virtual screening studies. These calculations were intended to complement the molecular docking and dynamics analyses by providing a deeper understanding of the intrinsic electronic characteristics that govern ligand stability and reactivity. In particular, density functional theory (DFT) was applied to elucidate how electronic distribution and reactivity patterns might influence the observed binding behavior and overall chemical robustness of the lead molecules. The geometries of the ligands were first optimized using the Jaguar module within the Schrödinger Suite (Schrödinger Release 2024-1), employing DFT with the B3LYP hybrid functional and the 6-31G(d,p) basis set (40–43). The highest occupied molecular orbital (HOMO) and lowest unoccupied molecular orbital (LUMO) energies were determined to estimate the ligands’ chemical reactivity and kinetic stability. The energy difference between HOMO and LUMO (HOMO-LUMO gap) was calculated, providing insight into the electron excitation and potential reactivity of the compounds. Average local ionization energy (ALIE) maps were computed within the Jaguar module to identify the regions of each molecule most susceptible to electrophilic attack. The ALIE was visualized as a surface overlay, highlighting the areas of highest and lowest ionization potential across the molecular surface. Regions with lower ALIE values correspond to sites of lower ionization energy, highlighting chemically reactive sites with weakly bound electrons. Electrostatic potential (ESP) maps were generated to analyze the charge distribution on the molecular surface, enabling the identification of electron-rich and electron-deficient regions that may interact with the protein’s active site. The ESP was calculated using the DFT/B3LYP/6-31G** level of theory, ensuring a reliable representation of the electrostatic environment (44).

### Recombinant protein production and purification

The pET28a-PtpB-Mtb construct was transformed into *E. coli* BL21(DE3) and cultured in LB medium with 50 µg/mL kanamycin at 37°C to an OD_600_ of 0.5. Overexpression of the His-tagged recombinant proteins was induced with 0.1 mM isopropyl β-D-1-thiogalactopyranoside at 20°C for 20 h. Cells were harvested, and the expression of recombinant protein was analyzed using SDS/PAGE. The harvested cell pellet was lysed in Tris-NaCl buffer (pH 7.4, 1 mM PMSF) and sonicated (32 Hz, 15 min, 10 s on/off cycle at 4°C). The lysate was clarified by centrifugation. The supernatant, containing the His-tagged PtpB-Mtb, was purified via Ni-NTA affinity chromatography. The filtered supernatant was loaded onto a pre-equilibrated column (Tris-NaCl buffer pH 7.4) and washed (Tris-NaCl buffer pH 7.4, 30 mM imidazole). Protein elutions were performed using a 50–200 mM imidazole gradient and analyzed on SDS/PAGE. Fractions containing single bands were pooled, and the sample was dialyzed at 4°C using SnakeSkin 10 kDa cut-off dialysis tubing. The purified PtpB-Mtb protein was stored in 20% glycerol at –20°C, and its phosphatase activity was validated using pNPP as substrate, in accordance with established protocols for mycobacterial PTPB inhibitor studies. The purified protein was checked for its phosphatase activity using pNpp as substrate.

### Enzyme inhibition assay

The inhibitory action of test compounds against phosphatase activity of PtpB-Mtb was assessed in triplicates, in a 96-well microplate. Reactions (200 µL) contained 1.5 µg protein and 50 µM test compound in 50 mM Tris/100 mM NaCl buffer (pH 7.4). After pre-incubation (15 min, 37°C), 1.3 mM pNPP was added to the reaction mixture and incubated at 37°C for 30 min. Absorbance was measured at 415 nm in an iMark microplate absorbance reader (BioRad) to quantify p-nitrophenyl phosphate (pNPP) hydrolysis. Controls without enzyme were included to correct for non-enzymatic substrate hydrolysis.

### IC₅₀ and Kᵢ determination

Compounds exhibiting greater than 60% inhibition against PtpB-*Mtb* were further evaluated for IC₅₀ determination using twofold serial dilutions. Inhibition constants (Kᵢ) were determined by adding varying concentrations (0 µM, 10 µM, and 50 µM) of selected inhibitor compounds to the protein (PtpB-Mtb) to make a 200 µL reaction mixture containing (50 mM Tris/100 mM NaCl) with a pH 7.4. After a 15-min incubation at room temperature, pNPP was added. The reaction mixture was again incubated for 30 min at 37°C, following which absorbance was recorded at 415 nm in an iMark Microplate absorbance reader (BioRad). Dose–response data were fitted to a non-linear regression curve (inhibitor vs response) in GraphPad Prism to determine IC_50_ values of each compound. The mode of inhibition was analyzed via Lineweaver–Burk plot, where the double reciprocal of inhibitor concentration was plotted against velocity. The inhibition constant (K_i_) was calculated from the x-intercept of the Lineweaver–Burk plots according to the equation for competitive inhibition, with values reported as mean ± SD from three independent experiments. All assays were performed in triplicate across three independent experiments, ensuring data robustness and reproducibility.

### Cytotoxicity evaluation using MTT assay

To assess the general cytotoxic profile of lead compounds, human embryonic kidney cells (HEK293T) were employed as representative mammalian cell models. HEK293T cells were maintained in Dulbecco’s Modified Eagle’s Medium supplemented with 10% heat-inactivated FBS, 2 mM L-glutamine, and 1% penicillin-streptomycin. The cell line was incubated at 37°C under 5% CO₂ and sub-cultured every 2–3 days using 0.25% trypsin-EDTA. Cells were harvested at sub-confluence and seeded into sterile 96-well flat-bottom microplates at a density of 2 × 10³ cells per well in 100 µL of respective culture medium. After 24 h of incubation to allow cell attachment and recovery, compounds 24807456 (D9) and 5347349 (D6) were added in serial dilutions prepared from 100 mM stock solutions in DMSO. Final compound concentrations ranged from 100 µM to 0.0003 µM, with DMSO concentration maintained below 0.1% (vol/vol) to avoid solvent-induced cytotoxicity. Cells were incubated with compounds for 48 or 72 h, with incubation duration optimized to minimize media evaporation and maintain cell viability. Following incubation, 50 µL of MTT reagent (2 mg/mL in sterile phosphate-buffered saline) was added to each well, and plates were incubated for 2 h at 37 °C under 5% CO₂. Subsequently, the culture medium was aspirated, and the formazan crystals formed by metabolically active cells were solubilized by adding 150 µL of DMSO per well. Plates were gently vortexed to ensure complete dissolution, and absorbance was measured at 540 nm using a Molecular Devices SpectraMax M5 microplate reader. Cell viability was calculated as a percentage relative to untreated control wells. Dose–response curves were generated by plotting cell viability against the negative logarithm of compound concentrations using GraphPad Prism 9.0 software. Half-maximal inhibitory concentration (IC₅₀) values were derived through nonlinear regression analysis (45). All experiments were performed in quadruplicate to ensure reproducibility. Compounds were considered cytotoxic if cell viability fell below 70%, consistent with prior cytotoxicity thresholds reported for PtpB-Mtb inhibitors.

### Biolayer interferometry kinetic analysis

Binding kinetics between PtpB-*Mtb* and the identified lead compounds were analyzed using a Sartorius BLI Octet R4 system with dip and read Ni-NTA biosensors pre-immobilized with novel nickel charged Tris-NTA (46). The biosensors were equilibrated in sample dilution buffer (1× PBS, pH 7.4 with 0.01% Tween-20 and 0.1%). 100 µg/mL His-tagged PtpB-Mtb was loaded and immobilized by establishing a stable baseline for 60 seconds, followed by a buffer wash. The association/dissociation kinetics were measured across a concentration range of 0–100 µM of inhibitors in buffer (1× PBS, pH 7.4 with 0.01% Tween-20 and 0.1% BSA) with a 720–900 s association and 300 s dissociation. Data fitting (*R*² > 0.9) was performed using Octet software, providing quantitative insights into binding affinities and kinetic profiles.

## RESULTS

### Validation of the docking protocol

**Fig 1:**
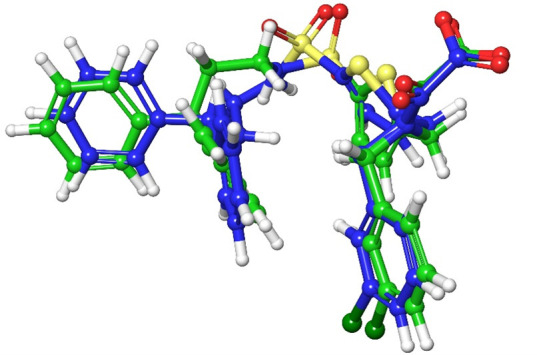
Validation of docking using retro validation—OMTS docked pose (green) and co-crystallized pose (blue).

To assess the reliability of the molecular docking strategy employed, both root mean square deviation (RMSD) and enrichment analyses were performed. The accuracy of the docking method was evaluated by re-docking the co-crystallized ligand OMTS and comparing its predicted binding conformation to the experimentally resolved structure. The resulting RMSD value was 1.3397 Å, indicating a high degree of structural similarity between the docked and reference poses. This close alignment supports the robustness of the docking protocol and confirms its suitability for subsequent virtual screening experiments. The superimposition of the docked pose of OMTS (shown in green) with the co-crystallized pose (shown in blue) demonstrates the accurate reproduction of the binding mode Fig. 1.

**Fig 2:**
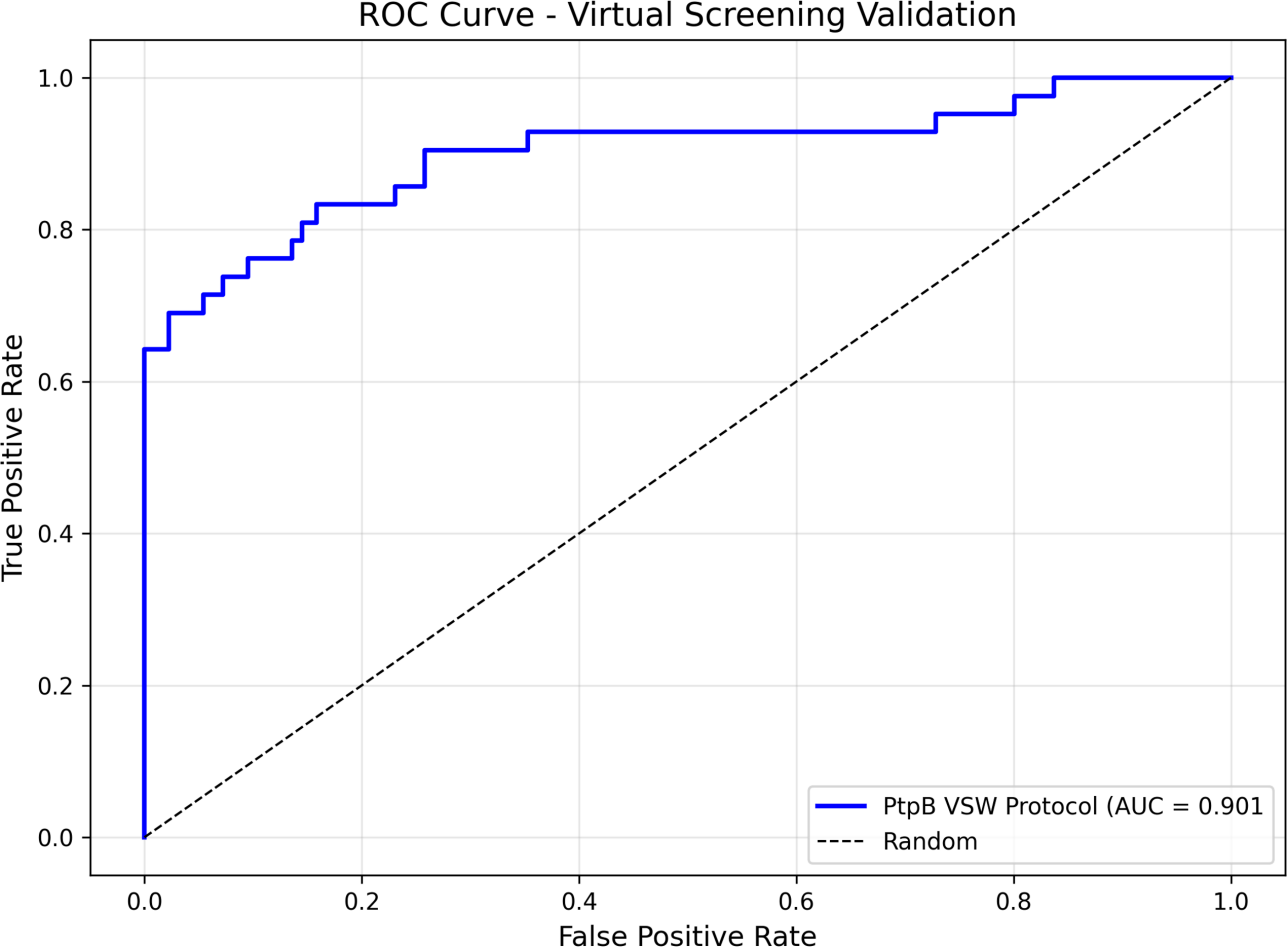
ROC curve for docking protocol validation-ROC curve demonstrating the screening performance of the Glide XP docking protocol. The curve plots true-positive rate (sensitivity) against false-positive rate (1-specificity) for discrimination between known PtpB-Mtb inhibitors and decoy compounds. The AUC of 0.901 indicates excellent screening performance, substantially exceeding random selection (diagonal dashed line, AUC = 0.5). This high discriminative power validates the protocol’s capability to prioritize active compounds in prospective virtual screening campaigns.

To evaluate the protocol’s effectiveness in distinguishing active compounds from inactive molecules, retrospective enrichment analysis was conducted using a curated validation data set. ROC curve analysis yielded an AUC value of 0.901 (Fig. 2), indicating outstanding discrimination between known PtpB-Mtb inhibitors and decoy compounds. This exceptional performance significantly exceeds the random selection baseline (AUC = 0.5) and demonstrates that the docking methodology reliably prioritizes active compounds over inactive molecules across the scoring spectrum.

The high AUC value validates the protocol’s utility for prospective virtual screening applications and provides confidence that top-ranked compounds from the ChemBridge library screening represent genuine hits rather than false positives. These comprehensive validation metrics, combining both pose prediction accuracy and screening performance assessment, establish a robust foundation for the identification of novel PtpB-Mtb inhibitors.

### Virtual screening identifies lead pyrazolo[4,3-c]pyridine/diazepane derivatives targeting PtpB-Mtb

**Fig 3:**
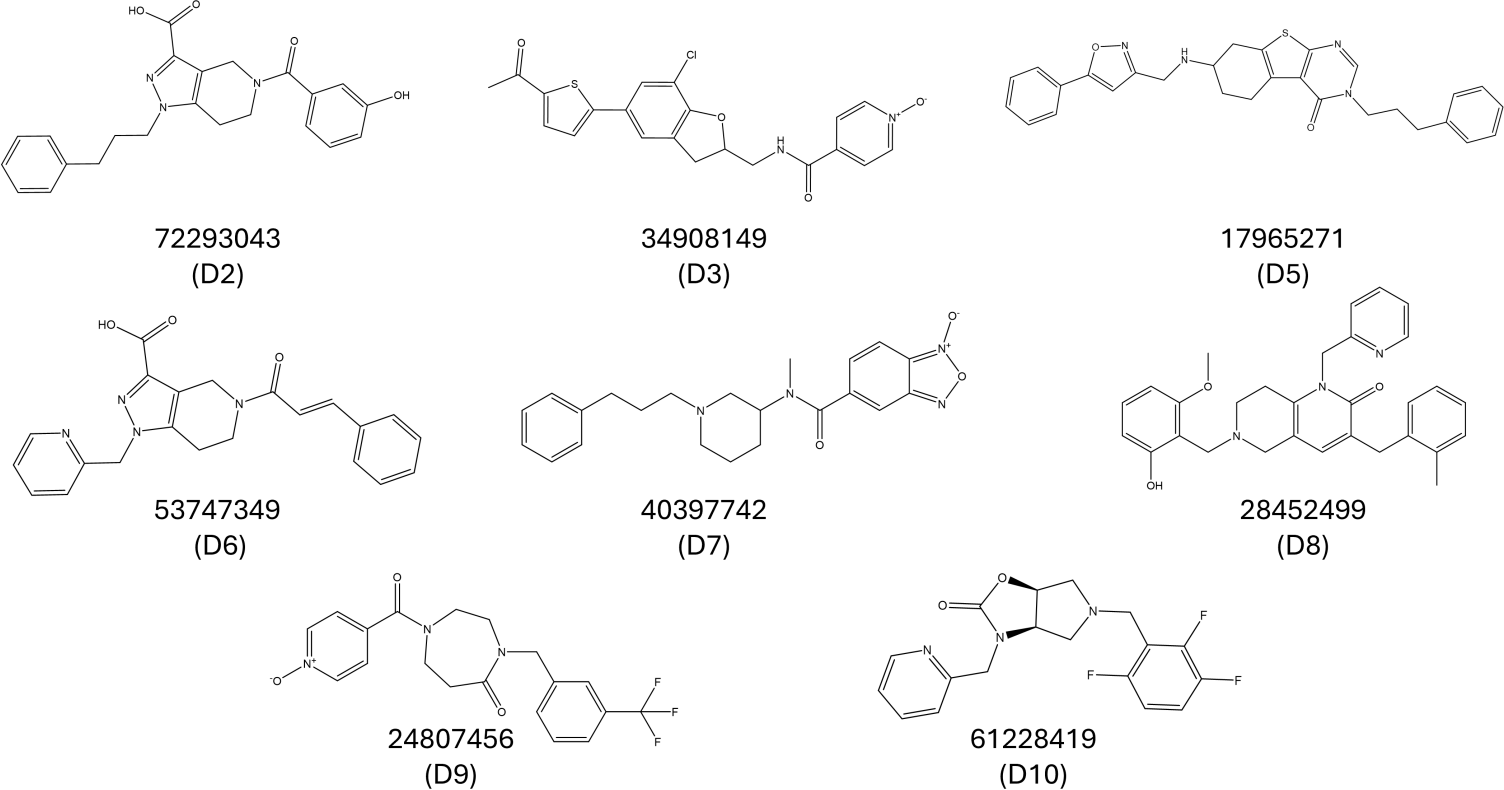
Virtual screening of the ChemBridge compound library against Mtb-PtpB. A panel of eight compounds (D2, D3, and D5–D10) with high binding affinity toward the target protein was selected. The structures shown represent the top-ranking candidates prioritized for further *in vitro* validation based on binding interactions, drug-likeness, and predicted pharmacokinetic properties.

Focused virtual screening of the ChemBridge compound library against the active site of PtpB-Mtb yielded eight top-ranking candidates based on docking scores and MMGBSA binding energy calculations (Table 1). For benchmarking, the co-crystallized ligand OMTS was also evaluated, exhibiting a docking score of –11.697 kcal/mol and an MMGBSA binding energy of –87.37 kcal/mol. Among the shortlisted hits, docking scores ranged from –9.394 to –12.070 kcal/mol, while MMGBSA energies ranged from –38.68 to –81.67 kcal/mol. Several compounds, including D7 (–12.070 kcal/mol) and D3 (–11.551 kcal/mol), displayed docking scores comparable to OMTS, whereas D8 (–81.67 kcal/mol) and D5 (–77.34 kcal/mol) approached OMTS in MMGBSA binding energy. Compound 24807456 (D9), a 1-(1-oxidoisonicotinoyl)−4-[3-(trifluoromethyl)benzyl]−1,4-diazepan-5-one, exhibited a favorable docking score of –11.427 kcal/mol, while compound 53747349 (D6), a 5-[(2E)−3-phenylprop-2-enoyl]−1-(pyridin-2-ylmethyl)−4,5,6,7-tetrahydro-1H-pyrazolo[4,3-c]pyridine-3-carboxylic acid, showed a favorable MMGBSA binding energy of –71.63 kcal/mol. Notably, both compounds were also found to possess unique chemical scaffolds, highlighting their potential as promising hits. Overall, all eight compounds demonstrated promising predicted binding profiles relative to OMTS, supporting the robustness of the screening protocol. The chemical structures of all eight selected compounds are summarized in Fig. 3.

**TABLE 1:** Binding affinity and binding energy of the top eight hit compounds targeting *Mtb* PtpB

Sr. no	Lab code	Compound code from ChemBridge library	Binding affinity (−kcal/mol)	Binding energy (−kcal/mol)	IUPAC name
1.	D7	40397742	−12.070	−52.50	N-methyl-N-[1-(3-phenylpropyl)−3-piperidinyl]−2,1,3-benzoxadiazole-5-carboxamide 1-oxide
2.	D3	34908149	−11.551	−58.94	N-{[5-(5-acetyl-2-thienyl)−7-chloro-2,3-dihydro-1-benzofuran-2-yl]methyl}isonicotinamide 1-oxide
**3.**	D9	24807456	−11.427	−38.68	1-(1-oxidoisonicotinoyl)−4-[3-(trifluoromethyl)benzyl]−1,4-diazepan-5-one
4.	D10	61228419	−10.393	−51.13	(3aS*,6aR*)−3-(2-pyridinylmethyl)−5-(2,3,6-trifluorobenzyl)hexahydro-2H-pyrrolo[3,4-d] (1, 3)oxazol-2-one
5.	D2	72293043	−9.937	−73.46	5-(3-hydroxybenzoyl)−1-(3-phenylpropyl)−4,5,6,7-tetrahydro-1H-pyrazolo[4,3-c]pyridine-3-carboxylic acid
6.	D8	28452499	−9.715	−81.67	6-(2-hydroxy-6-methoxybenzyl)−3-(2-methylbenzyl)−1-(2-pyridinylmethyl)−5,6,7,8-tetrahydro-1,6-naphthyridin-2(1H)-one
7.	D5	17965271	−9.555	−77.34	7-{[(5-phenyl-3-isoxazolyl)methyl]amino}−3-(3-phenylpropyl)−5,6,7,8-tetrahydro (1)benzothieno[2,3-d]pyrimidin-4(3H)-one
**8.**	D6	53747349	−9.394	−71.63	5-[(2E)−3-phenylprop-2-enoyl]−1-(pyridin-2-ylmethyl)−4,5,6,7-tetrahydro-1H-pyrazolo[4,3-c]pyridine-3-carboxylic acid
9.	OMTS	–^*a*^	−11.697	−87.37	O-methyl-thiosalicylate (co-crystallized ligand)

^*a*^ –, not applicable. OMTS is a co-crystallized ligand with *Mycobacterium tuberculosis* protein tyrosine phosphatase B (PtpB), retrieved from the Protein Data Bank (PDB ID: 2OZ5). The other compounds in the table were purchased from the ChemBridge library with their specific compound codes.

### ADME profiling of lead compounds

The QikProp module of Schrödinger Suite (Release 2024-1) was employed to evaluate key pharmacokinetic parameters for eight novel derivatives (D2, D3, D5, D6, D7, D8, D9, and D10) (Table 2). All compounds exhibited molecular weights within the optimal range for oral bioavailability (363.3–496.0 g/mol), with none exceeding the 500 g/mol threshold. Hydrogen bond donors (0–2) and acceptors (4–6) adhered to Lipinski’s criteria, ensuring compatibility with passive absorption mechanisms. Topological polar surface area (TPSA) values ranged from 45.67 Å² (D10) to 95.66 Å² (D2), remaining well below the 120 Å² limit associated with favorable membrane permeability. Aqueous solubility (LogSw) predictions ranged from −7.412 (D5) to −1.564 (D10), with all values above the −6.5 threshold, indicating adequate solubility for preclinical testing. Partition coefficients (cLogP) varied between 1.17 (D6) and 4.76 (D5), with seven compounds falling within the recommended range (−2.0 to 5.0). While D5 (cLogP = 4.76) marginally approached the upper limit, its solubility (−7.412 LogSw) remained within permissible bounds. Rotatable bond counts (3–8) for all derivatives were below the <10 cutoff, suggesting conformational flexibility conducive to oral bioavailability without compromising metabolic stability. D10 demonstrated the lowest TPSA (45.67 Å²) and rotatable bonds (4), suggesting enhanced membrane permeability. Conversely, D5 exhibited the highest molecular weight (496.0 g/mol) and lipophilicity (cLogP score of 4.76), indicating scope for minor structural refinements to improve solubility and pharmacokinetic balance. Collectively, six derivatives (D2, D3, D6, D7, D9, and D10) met all optimal ADMET criteria, while D5 and D8 (cLogP = 3.97) may require minor optimizations to balance solubility and permeability. These results validate pyrazolo[4,3-c]pyridine and 1,4-diazepane scaffold as a promising foundation for developing orally bioavailable PtpB-Mtb inhibitors.

**TABLE 2:** Molecular descriptors of identified hits

COMPOUND_ID of ChemBridge libary	Molecular weight	H-bond donor	H-bond acceptor	cLogP	Log Sw	Total polar surface area	Rotatable bonds
72293043 (D2)	405.5	2	5	2.02	−3.301	95.66	6
34908149 (D3)	428	1	5	2.98	−7.171	82.34	5
17965271 (D5)	496	1	5	4.76	−7.412	72.95	8
53747349 (D6)	388.4	1	5	1.17	−1.731	88.32	5
40397742 (D7)	394	0	6	3.228	−3.346	76.52	6
28452499 (D8)	481	1	5	3.97	−4.758	67.59	6
24807456 (D9)	393	0	4	2.87	−3.243	67.56	3
61228419 (D10)	363.3	0	4	2.391	−1.564	45.67	4
Optimum range (hit/lead)	130.0–500.0	0.0–5.0	2.0–10.0	−2.0 to 5.0	−6.5 to 0.5	7.0–200.0	<10

Although all derivatives displayed favorable pharmacokinetic characteristics, compound D5 showed relatively higher lipophilicity and lower solubility compared with the others. These remain within acceptable limits but could be improved through targeted structural optimization to enhance overall drug-like balance.

To contextualize the translational potential of compounds D6 and D9, their predicted ADMET profiles were compared with those of established first-line anti-tubercular agents. Rifampin, despite its high molecular weight (823 Da) and TPSA (220 Å²), achieves excellent oral bioavailability (~95%), demonstrating that TB drugs can succeed even when exceeding typical drug-likeness thresholds. In contrast, the identified compounds exhibit molecular weights (388–393 Da) and TPSA values (67.6–88.3 Å²) well within optimal ranges, comparing favorably to isoniazid (137 Da, 68 Å²), ethambutol (204 Da, 58 Å²), and pyrazinamide (123 Da, 69 Å²).

The moderate lipophilicity of D6 (cLogP = 1.17) and D9 (cLogP = 2.87) may provide an advantage over highly hydrophilic first-line drugs. While isoniazid (cLogP = −0.7) and ethambutol (cLogP = −0.3) exhibit minimal lipophilicity that can limit tissue penetration, D6 and D9 achieve a favorable balance that may support both aqueous solubility and membrane permeability. This property is particularly relevant for accessing intracellular mycobacteria within macrophages and granulomatous lesions where TB persists.

Based on their physicochemical profiles, oral bioavailability of 75%–85% is predicted for both compounds—comparable to ethambutol and approaching that of isoniazid and pyrazinamide. The overall ADMET features suggest these scaffolds are suitable for oral formulation, a critical factor given the prolonged treatment duration required for TB therapy. Experimental pharmacokinetic validation, however, will be essential to confirm that these predicted properties translate into favorable *in vivo* exposure and tissue distribution.

### MD simulation of compounds D6 (53747349), D9 (24807456), and OMTS

Compounds D6 (53747349) and D9 (24807456) were chosen for extended 500 ns MD simulations based on their favorable ADMET profiles and promising docking scores. These simulations aimed to evaluate the dynamic stability and detailed binding interactions of each ligand within the PtpB active site, providing complementary insights beyond static docking models.

**Fig 4:**
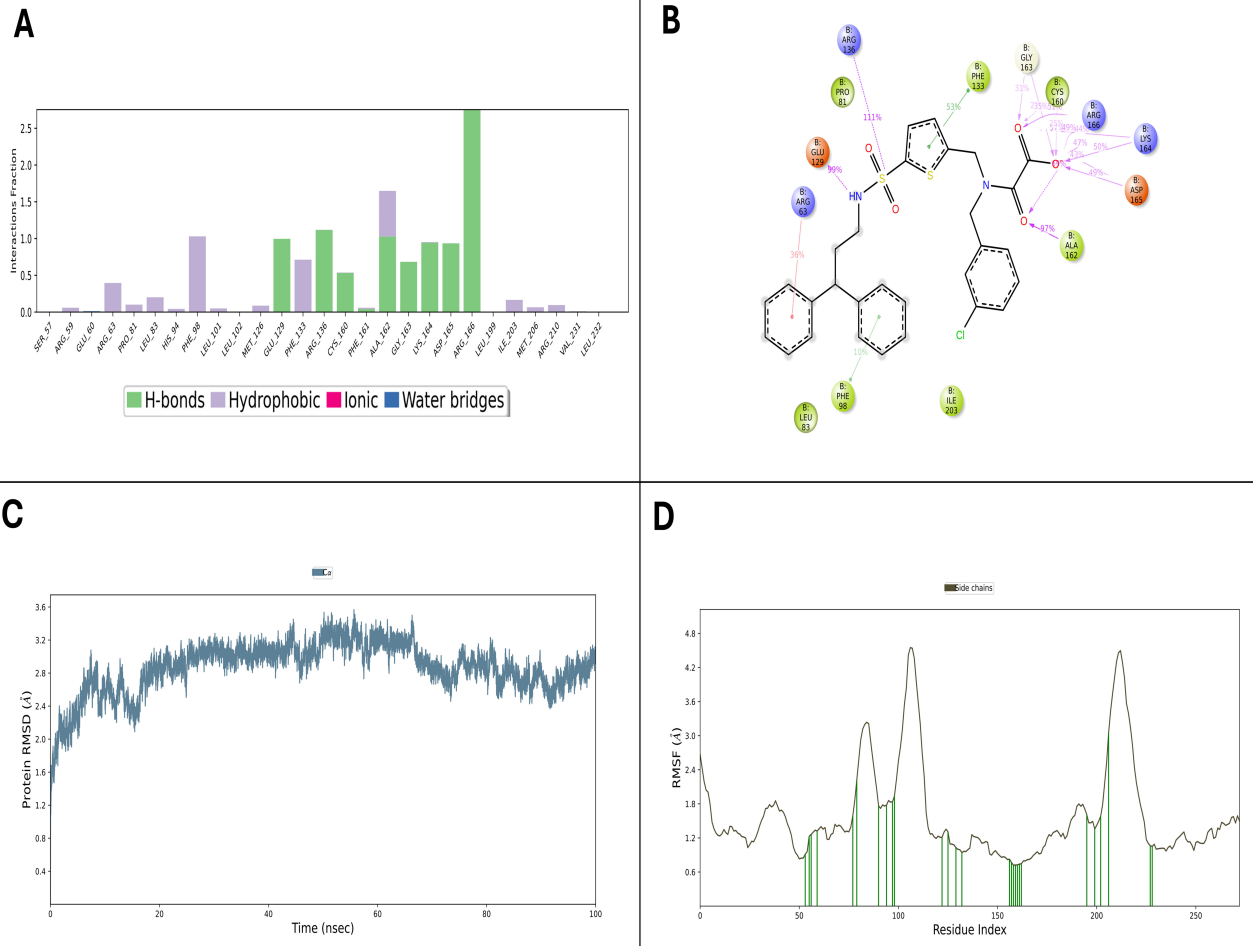
MD simulation analysis of the OMTS–PtpB complex. (**A**) Representative 3D interaction snapshot showing key hydrogen bonds, hydrophobic, and ionic contacts formed between OMTS and the binding site residues. (**B**) 2D interaction diagram depicting persistent interactions with critical residues such as Asp165, Lys166, and Glu62 throughout the 100 ns trajectory. (**C**) RMSD plot of the protein backbone indicating complex stability over time. (**D**) RMSF plot illustrating residue-level flexibility, with limited mobility near the active site and higher fluctuations at peripheral loops.

OMTS, a known inhibitor, was subjected to a 100 ns MD simulation to establish a reliable benchmark. Despite the shorter timescale, OMTS maintained key interaction patterns with active site residues ASP165, LYS166, and GLU62, forming stable hydrogen bonds and hydrophobic contacts throughout the simulation (Fig. 4A and B). The protein backbone RMSD remained confined to a narrow 2.0–3.2 Å range (Fig. 4C), signaling overall structural stability comparable to that observed in the test compounds. RMSF analysis (Fig. 4D) further confirmed limited residue flexibility, particularly within the binding site.

**Fig 5:**
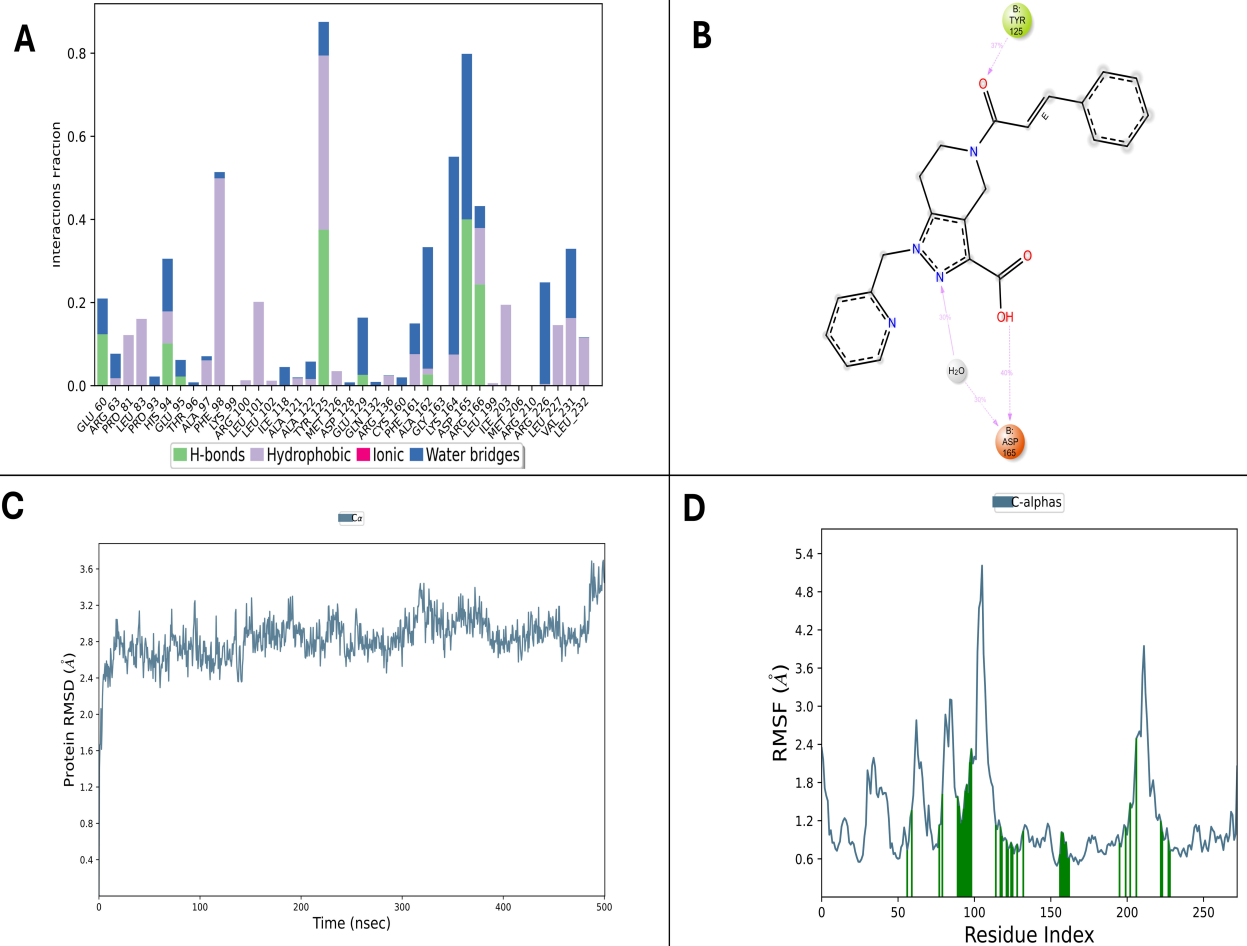
MD simulation analysis of compound 53747349 (D6) in complex with PtpB-Mtb over 500 ns. (**A**) Interaction profile histogram showing the fraction of simulation time each interaction type was maintained. (**B**) 2D ligand interaction diagram with key active site residues ASP165 and TYR125. (**C**) Protein backbone RMSD trajectory demonstrating complex equilibration and stability. (**D**) Per-residue RMSF values highlighting rigid binding site residues versus flexible peripheral loops. The complex maintained ≥7 simultaneous contacts throughout the simulation.

The 500 ns MD simulation of D6 revealed rapid equilibration within the first 50 ns, with protein backbone RMSD oscillating between 1.5 and 3.0 Å, underscoring minimal perturbation of global protein conformation (Fig. 5C). Ligand RMSD values also remained consistently low, reflecting the ligand’s firm positioning within the active site. The ligand’s Rg and solvent-accessible surface area indicated sustained ligand compactness and stability across the trajectory. Additionally, RMSF analysis revealed limited flexibility at key binding site residues such as ASP165, LYS166, and GLU60, emphasizing their crucial role in stabilizing the ligand–protein interaction (Fig. 5D).

Fluctuations observed were largely confined to solvent-exposed loop regions, away from the binding pocket, reinforcing the ligand’s stabilizing influence without compromising structural integrity. Throughout the simulation, hydrogen bonds involving ASP165, LYS164, and GLU60 dominated the protein–ligand contacts. Complementary hydrophobic interactions with residues like PHE98, LEU102, and VAL231 supported the affinity, while intermittent ionic contacts and consistent water-mediated bridges involving ARG166 and ASP164 enriched the interaction network. Tyr125 contributed an additional polar interaction that remained stable for around 37% of the trajectory (Fig. 5A and B). The complex maintained at least seven simultaneous contacts for most of the simulation, indicating a persistent and robust binding mode. Additionally, the involvement of a bridging water molecule indicates that the hydrogen-bonding network within the binding pocket was flexible yet consistently maintained, helping to anchor the ligand in a favorable orientation throughout the simulation period.

**Fig 6:**
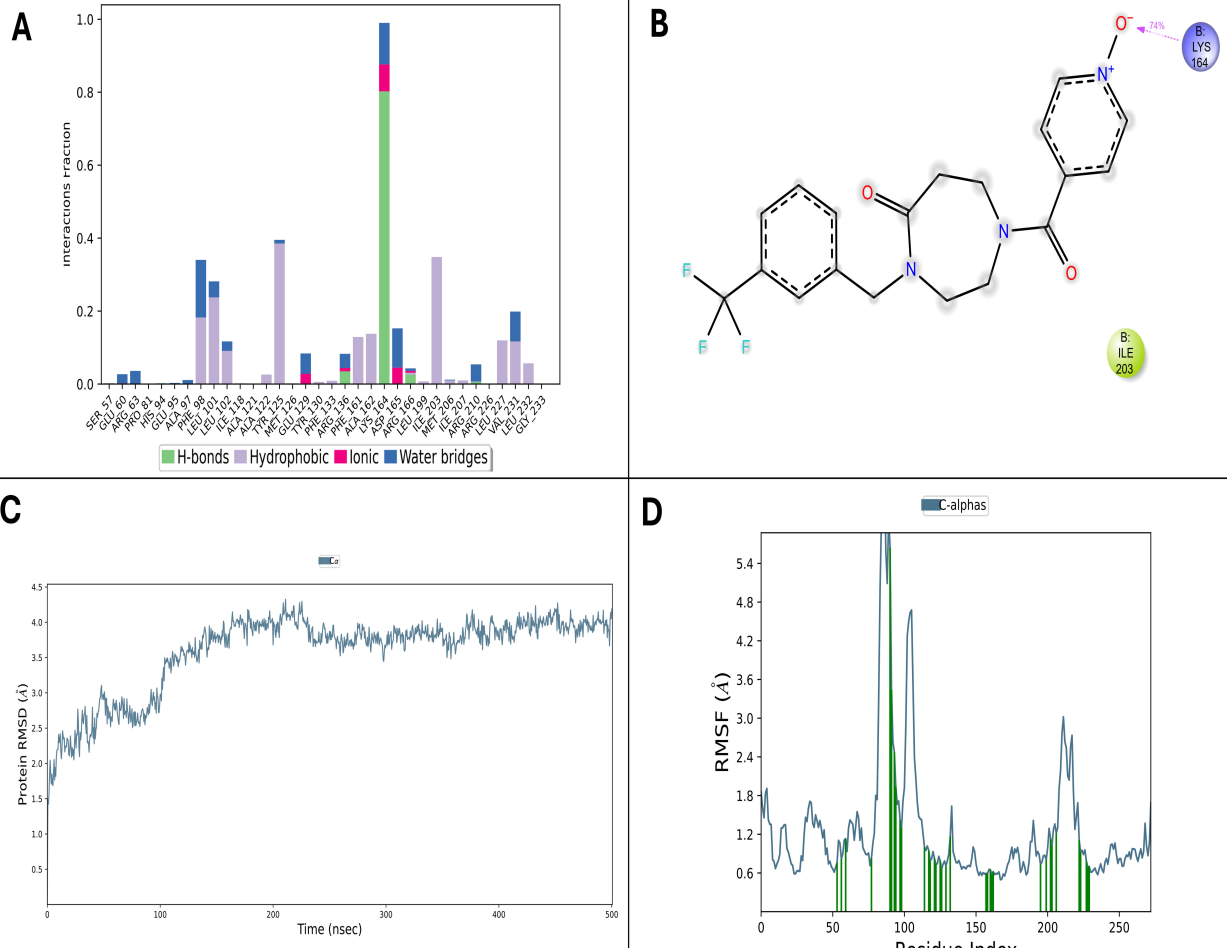
MD simulation analysis of compound 24807456 (D9) in complex with PtpB-Mtb over 500 ns. (**A**) Interaction profile histogram showing sustained hydrogen bonding and hydrophobic contacts. (**B**) 2D ligand interaction diagram illustrating engagement with catalytic residues LYS164 and ILE203. (**C**) RMSD plot indicating structural stability with fluctuations between 1.5 and 3.5 Å. (**D**) RMSF analysis confirming limited active site flexibility. The complex maintained ≥7 simultaneous contacts throughout the trajectory.

Similarly, D9 also demonstrated a stable association with PtpB-Mtb throughout a 500 ns MD simulation. The protein backbone RMSD equilibrated quickly and fluctuated mainly between 1.5 and 3.5 Å (Fig. 6C), confirming that the protein structural framework remained stable. Ligand RMSD and Rg were also found to remain steady, highlighting sustained ligand conformational stability. RMSF measures further indicated rigidity of essential active-site residues ASP165, LYS164, and GLU60, while enhanced flexibility was mostly evident in distal loops, suggesting that ligand binding preserved the protein’s structural core (Fig. 6D). Persistent hydrogen bonds with these same critical residues stabilized the ligand throughout. Hydrophobic interactions with residues such as PHE98, LEU101, TYR125, and ILE203 were consistently observed, along with water bridges involving ARG166 and ARG136, which provided additional stabilization. Along with the dominant hydrogen-bond interaction with Lys164 that persisted for about 74% of the trajectory, D9 also exhibited hydrophobic stabilization through Ile203. Although ionic interactions were less frequent, they complemented the binding network when present (Fig. 6A and B). Moreover, multiple contacts were maintained continuously across the trajectory, underscoring a firm and specific ligand binding.

Collectively, these MD simulations confirm that both D6 and D9 maintain key pharmacophoric interactions with PtpB, including stable hydrogen bonding to catalytic residues and persistent hydrophobic contacts within the binding pocket. This multifaceted interaction profile underpins their binding site specificity and structural stability, supporting their potential as promising candidates for further inhibitor development.

**Fig 7:**
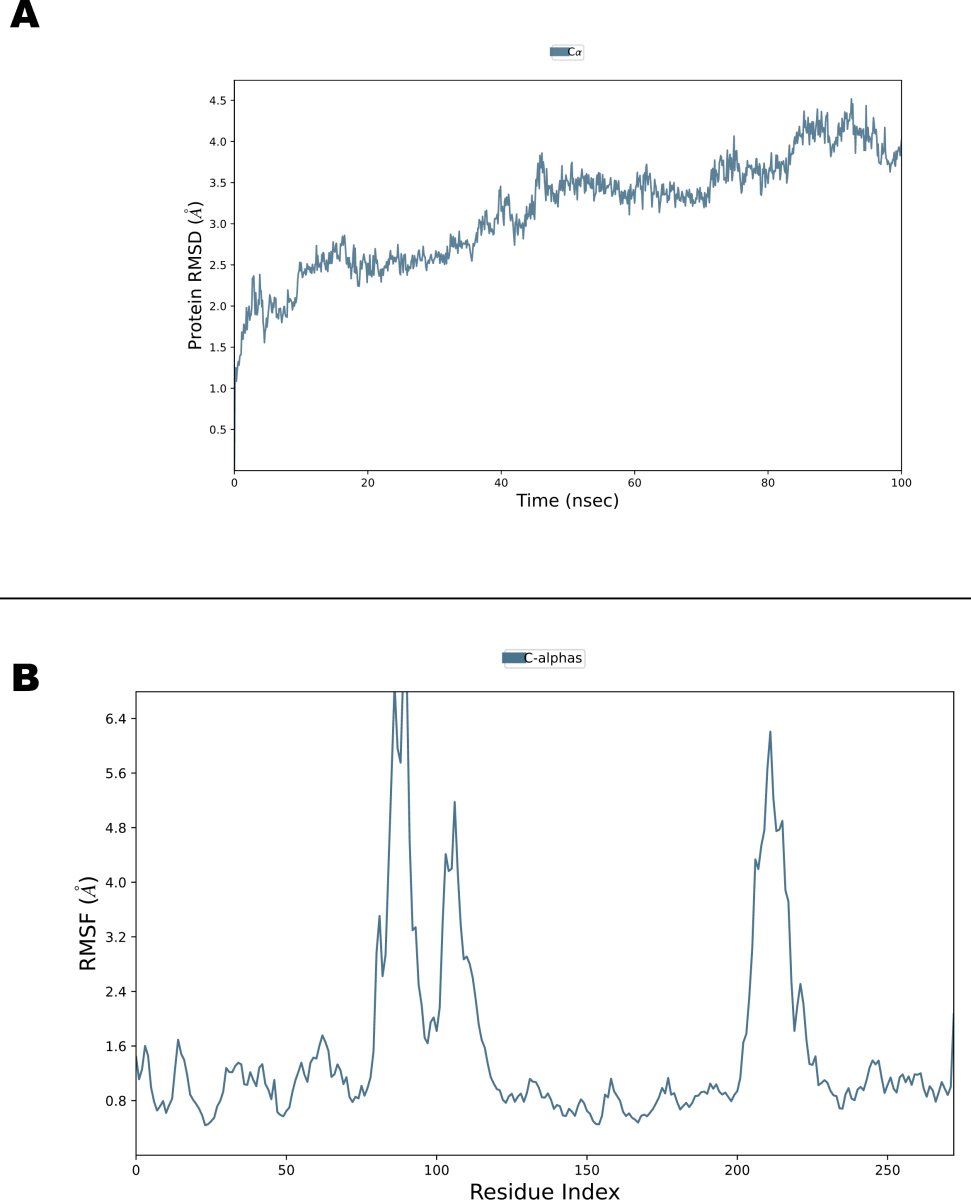
MD simulation analysis of apo-protein PtpB-Mtb. (**A**) RMSD trajectories of protein backbone comparing apo PtpB-Mtb with ligand-bound complexes over a 100 ns simulation. The apo protein shows higher RMSD fluctuations, indicating reduced structural stability in the absence of ligand. (**B**) RMSF profiles comparing residue flexibility between apo and ligand-bound states. Active site residues exhibit reduced flexibility upon ligand binding, demonstrating stabilization of the binding pocket region.

To evaluate ligand-induced stabilization, MD simulation of apo PtpB-Mtb was performed under identical conditions (Fig. 7). The apo protein exhibited higher backbone RMSD fluctuations compared to ligand-bound complexes, indicating reduced structural stability in the absence of ligand. Active site residues displayed elevated RMSF values in the apo form, demonstrating increased flexibility of the binding pocket when unoccupied. This comparison confirms that ligand binding stabilizes the active site region, reducing conformational dynamics and maintaining a more ordered binding pocket architecture. The enhanced rigidity of ligand-bound complexes validates that observed binding interactions contribute meaningfully to protein stabilization rather than reflecting inherent structural constraints.

**Fig 8:**
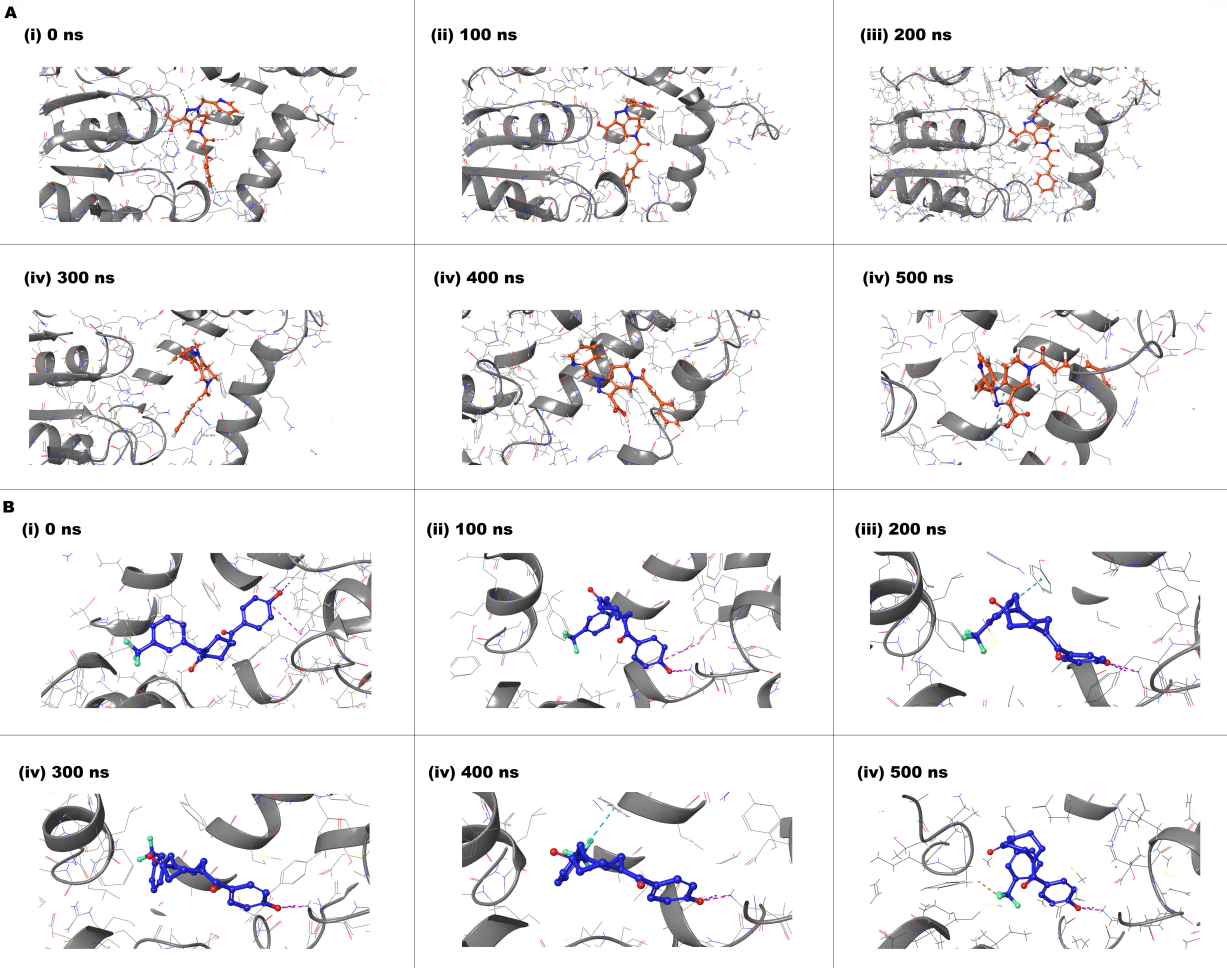
Representative snapshots of (**A**) compound D6 and (**B**) compound D9 in complex with PtpB-Mtb extracted at **(i)** 0, **(ii**) 100, **(iii)** 200, **(iv)** 300, **(v)** 400, and** (vi)** 500 ns of MD simulation in both (**A**) and (**B**). Ligands are shown in stick representation (colored by element: D6—orange and D9—blue), protein backbone is displayed as gray cartoon. All snapshots are shown from consistent viewing angles to facilitate comparison. The binding poses remain stable throughout the 500 ns trajectory, demonstrating persistent protein–ligand interactions.

Visual inspection of MD trajectories at representative timepoints (0, 100, 200, 300, 400, and 500 ns) revealed stable binding orientations for both compounds throughout the simulation period (Fig. 8). Compound D6 maintained consistent positioning within the active site with preservation of key hydrogen bonding interactions across all timepoints. Similarly, compound D9 exhibited stable binding with minimal conformational drift, demonstrating robust protein–ligand complex stability.

### Quantum chemical descriptor analysis

Quantum chemical descriptors were calculated to provide detailed insights into the electronic structure, stability, and reactivity of compounds D9 (24807456) and D6 (53747349) (Table 3), which are critical for understanding their behavior as potential inhibitors. Both compounds exhibited a comparable number of canonical orbitals (509 for D9 and 534 for D6), reflecting similar molecular complexity. Analysis of frontier molecular orbitals revealed that D6 possessed a slightly lower LUMO energy (−0.05641 Hartree) than D9 (−0.07125 Hartree), suggesting marginally enhanced electron affinity and a greater tendency to accept electrons, which can be advantageous for binding interactions with the target protein. The HOMO energies for both compounds were nearly identical (−0.22889 Hartree for D9, −0.22740 Hartree for D6), and the relatively narrow HOMO–LUMO gaps support the potential for favorable electronic transitions and chemical reactivity. Thermodynamic parameters further substantiated these findings: D6 exhibited higher entropy (146.52 cal/mol/K) and slightly greater enthalpy (13.58) compared to D9 (143.29 cal/mol/K and 13.13, respectively), indicating a marginally higher degree of molecular flexibility and thermodynamic favorability. Both compounds demonstrated negative free energy values (−30.10 for D6, −29.60 for D9), consistent with spontaneous and stable interactions under physiological conditions. The total internal energies at 298.15 K were closely aligned (−1294.76 au for D6, −1424.02 au for D9), while the mean ESP values were identical (0.001 kcal/mol for both), suggesting similar charge distributions on the molecular surface. Notably, D6 exhibited a slightly lower ALIE (204.30 kcal/mol) compared to D9 (209.77 kcal/mol), which may confer a subtle advantage in terms of chemical reactivity and potential for interaction with the protein active site.

**TABLE 3:** QM properties of hits 24807456 (D9) and 53747349 (D6) targeting *Mtb* PtpB

Properties	53747349 (D6)	24807456 (D9)
Docking score (kcal/mol)	−9.394	−11.427
MM-GBSA bind (kcal/mol)	−71.63	−38.68
Number of canonical orbitals	534	509
HOMO^a^ (Hartree)	−0.227400	−0.228890
LUMO^b^ (Hartree)	−0.056410	−0.071250
Entropy (cal mol⁻¹ K⁻¹, 298K)	146.522	143.285
Enthalpy (kcal mol⁻¹)	13.582	13.125
Free Energy (kcal mol⁻¹)	−30.103	−29.595
Total Internal Energy at 298.15K (atomic units [au])	−1294.757690	−1424.018220
ESP mean (kcal mol⁻¹)^c^	0.001	0.001
ALIE mean (kcal mol⁻¹)^d^	204.299	209.765

**Fig 9:**
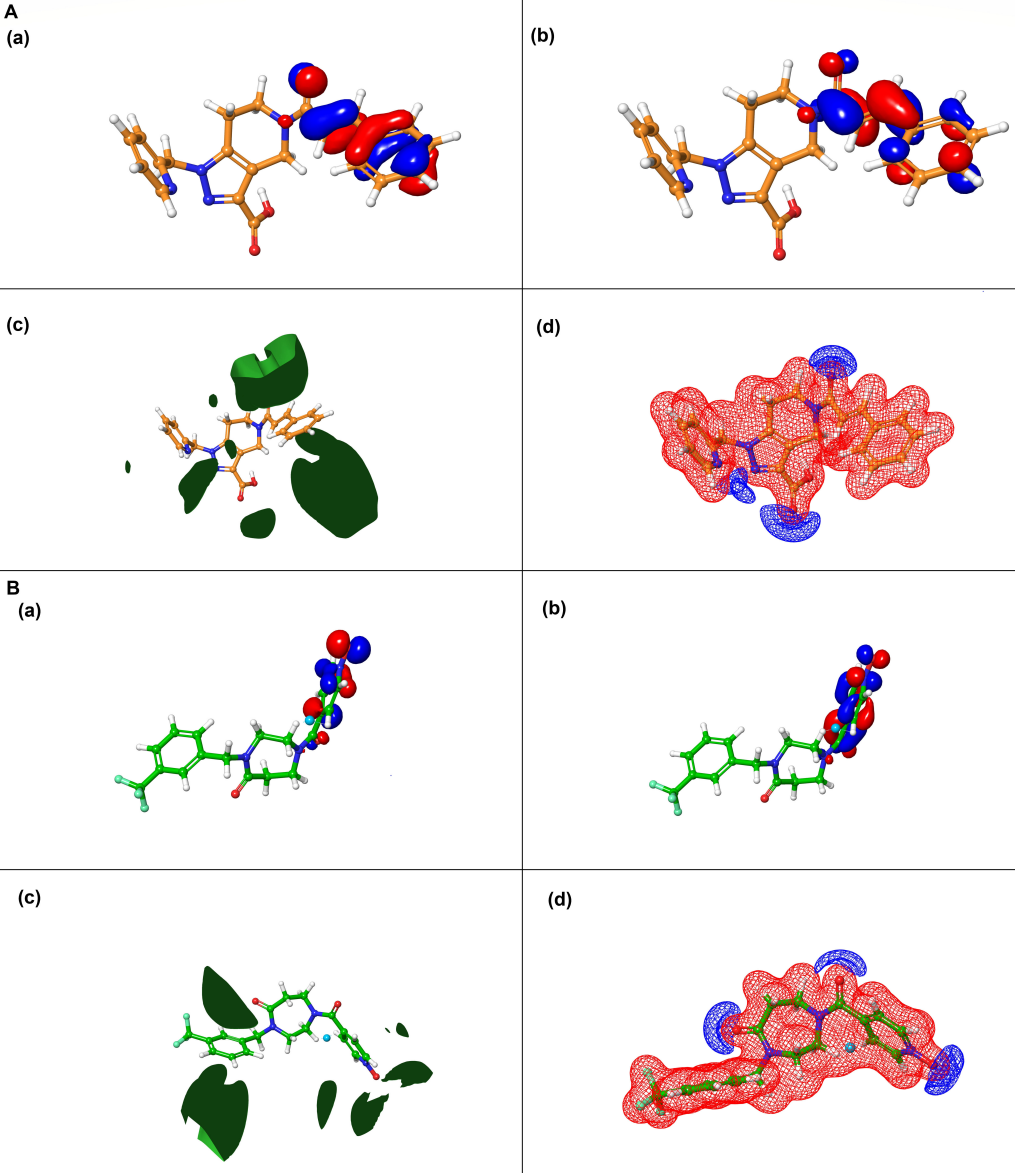
QM surface map analysis of compounds 53747349 (D6) and 24807456 (D9). DFT calculations were performed to explore the electronic characteristics of compounds (**A**) D6 and (**B**) D9. (**a**) and (**b**) display the HOMO and LUMO distributions, respectively, highlighting the regions involved in electron donation and acceptance. (**c**) illustrates the ALIE maps, indicating potential sites for electrophilic attack. (**d**) shows the ESP maps, with red and blue regions denoting areas of negative and positive potential, respectively. Together, these surface properties offer insight into the reactivity and interaction potential of the compounds with target biomolecules.

The electronic surface map analysis of both compounds (Fig. 9) further confirmed these observations. Figure 9A and B (a,b) depict the HOMO and LUMO distributions, respectively, highlighting regions on the pyrazolo[4,3-c]pyridine and diazepane moiety that may participate in electron donation and acceptance during binding. Panel 9A and B (c) shows the ALIE maps, which clearly identify potential electrophilic sites, aligning with the observed lower ALIE value of D6, supporting its slightly higher chemical reactivity. Panel 9A and B (d) presents the ESP maps, which reveal similar charge distributions but also highlight local regions of positive and negative potential that may influence binding orientation and interaction with the protein active site. These surface property visualizations complement the quantitative descriptors and provide a comprehensive picture of how electronic structure relates to inhibitory potential. Notably, regions with lower ALIE values correspond to sites characterized by reduced ionization potential, indicating zones of higher chemical reactivity. In the context of the PtpB-binding pocket, these low-ALIE regions suggest possible sites of enhanced interaction strength through charge transfer or hydrogen bonding with catalytic residues. The slightly lower ALIE value observed for D6 thus supports its greater electronic responsiveness, consistent with its stronger and more stable binding behavior in the enzyme active site.

These quantum chemical parameters not only offer a mechanistic understanding of the compounds’ electronic properties but also provide predictive value for their binding affinities and reactivity in the context of drug discovery, thereby guiding the selection and optimization of lead molecules. The favorable quantum profiles of both D6 and D9, particularly the slightly enhanced electron affinity and reactivity of D6, support their advancement as promising PtpB-*Mtb* inhibitor candidates.

### Inhibition of PtpB-*Mtb* activity and binding interactions of lead compounds

**Fig 10:**
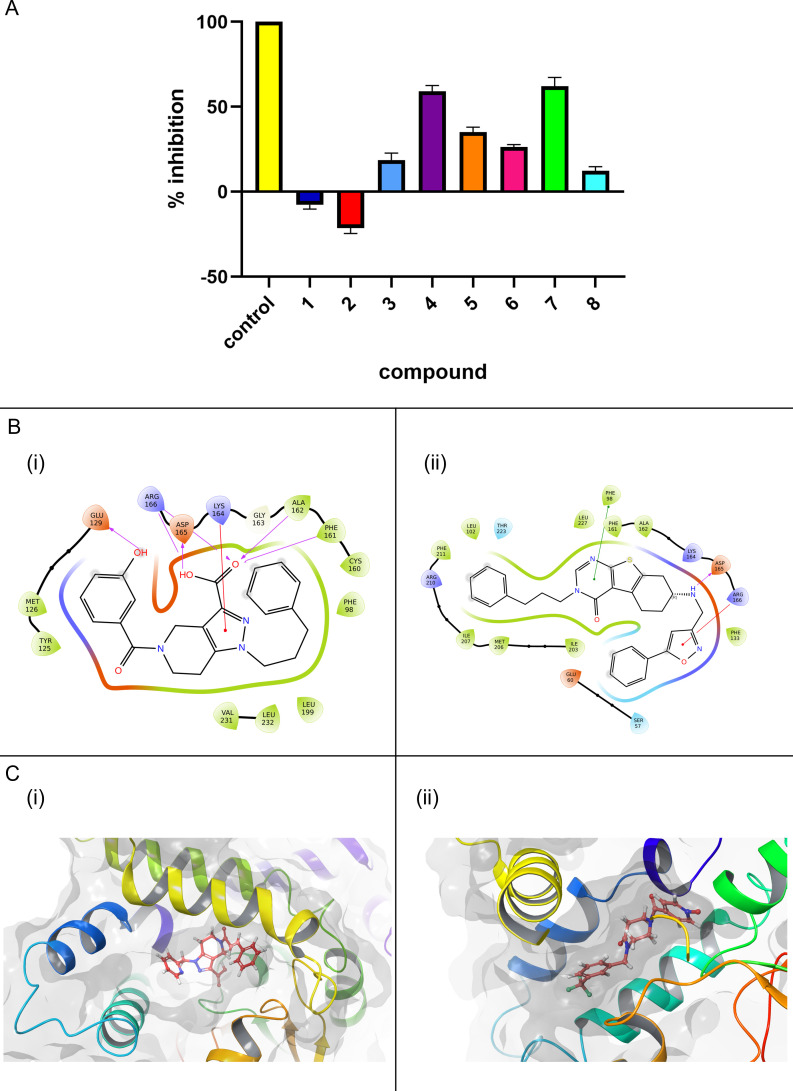
Inhibition profile, binding mode, and interaction pattern of top hits with Mtb-PtpB. (**A**) shows the inhibition of Mtb-PtpB activity using pNPP as the substrate. All candidates were tested at a concentration of 50 µM, among which compounds 125340 and 21595 exhibited the most significant inhibition. The ligands 53747349 (D6) and 24807456 (D9) are shown separately to highlight their respective interaction patterns with key active-site residues in panels **B(i)** and **B(ii)**, respectively. In **B(i)**, the ligand forms hydrogen bonds with GLU129, ASP165, and LYS164 and is surrounded by hydrophobic residues including PHE98, PHE161, CYS160, and ALA162. In **B(ii)**, the ligand forms hydrogen bonds with ASP165, ARG166, and LYS164 and engages in hydrophobic interactions with residues such as PHE98, PHE161, LEU227, and MET206. The presence of a sulfur atom in the ligand core may contribute to additional stabilizing interactions, and the extended hydrophobic tail enhances the overall binding affinity. The interactions suggest stabilization via hydrogen bonding and hydrophobic contacts within the binding pocket. Hydrogen bonds, hydrophobic interactions, and other critical contacts stabilizing the ligands within the binding pocket are indicated. The binding modes of compounds 53747349 (D6) and 24807456 (D9) within the active site of Mtb-PtpB are illustrated in panels **C(i)** and **C(ii)**, respectively. Color codes: Red arrows: hydrogen bonds, green arrows: π–π stacking or hydrophobic interactions, residue color: orange (acidic), blue (basic), green (hydrophobic), light blue (polar uncharged).

The inhibitory potential of the eight top-ranking compounds against PtpB-*Mtb* was evaluated using a pNPP enzymatic assay at a fixed concentration of 50 µM. As depicted in Fig. 10A, compounds 53747349 (D6) and 24807456 (D9) showed the most pronounced inhibition of phosphatase activity, with both exhibiting substantially higher inhibitory effects relative to the other candidates. The remaining compounds displayed variable and generally moderate levels of inhibition, while two candidates showed negligible or negative effects on enzyme activity.

To elucidate the molecular basis of inhibition, the binding interactions of compounds 53747349 (D6) and 24807456 (D9) within the PtpB-*Mtb* active site were analyzed. Two-dimensional interaction diagrams (Fig. 10B) revealed that both ligands formed extensive networks of hydrogen bonds and hydrophobic contacts with key catalytic residues. For compound 53747349 (D6) (panel B[i]), critical hydrogen bonds were observed with residues implicated in substrate recognition and catalysis, while hydrophobic interactions further stabilized the ligand within the binding pocket. Similarly, compound 24807456 (D9) (panel B[ii]) engaged in multiple polar and non-polar contacts, supporting its high inhibitory potency.

Three-dimensional representations of the ligand-bound complexes (Fig. 10C) provided further insight into the spatial orientation of the lead compounds within the enzyme’s active site. Both compounds were accommodated within the catalytic cleft, with their core scaffolds positioned to maximize interactions with essential residues. The conformational complementarity observed in these models is consistent with the strong inhibitory activity detected in the biochemical assays.

### Dose–response analysis and inhibition mechanism of hit compounds

**Fig 11:**
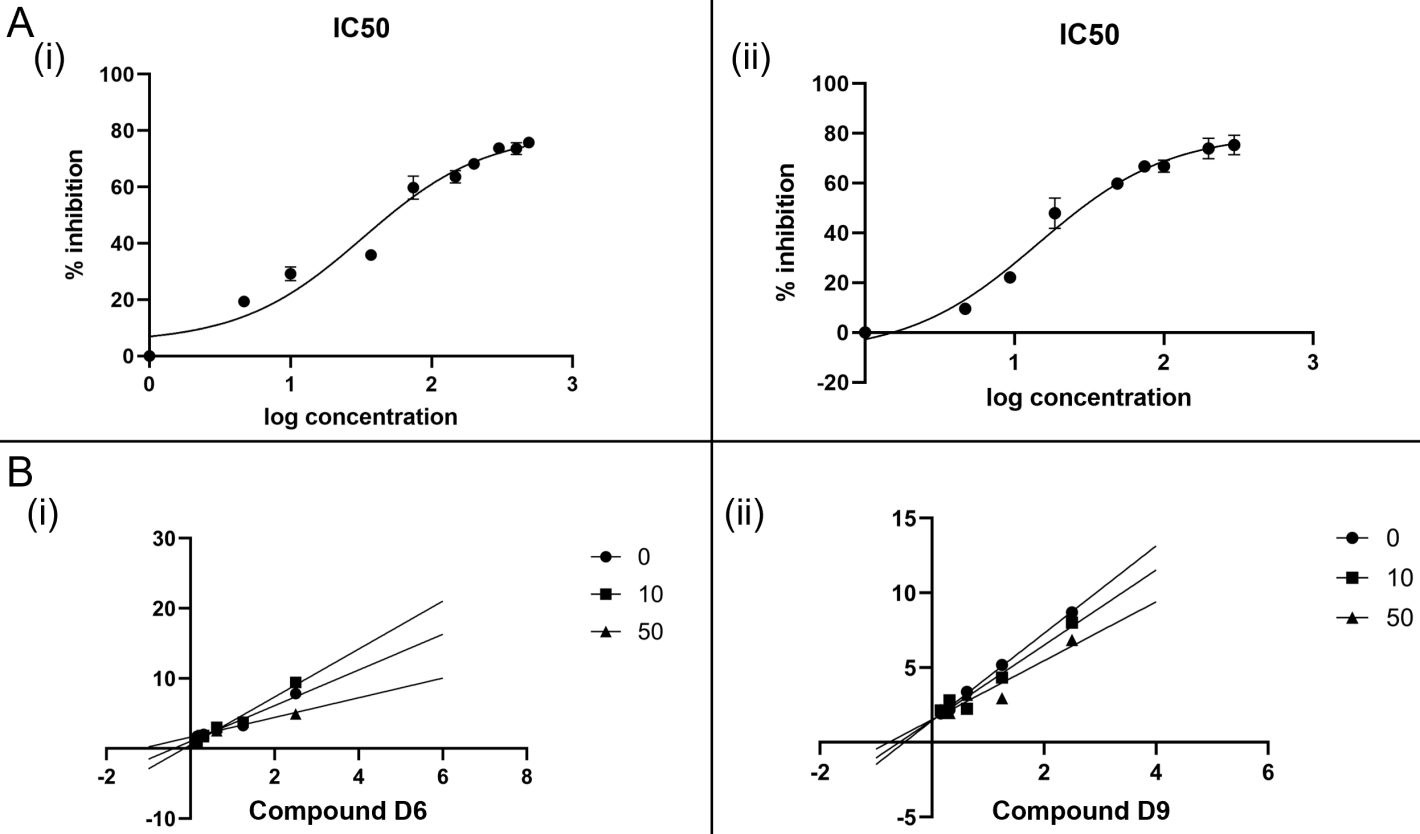
IC50 and type of inhibition calculations for identified inhibitors. (**A**) **(i)** Dose–response curve showing the half-maximal inhibitory concentration (IC₅₀) of compound D6 (53747349) on Mtb-PtpB activity, with an IC₅₀ of 32.62 ± 17.6 µM determined by nonlinear regression analysis (log[inhibitor] vs. response, three-parameter fit). (**A**) **(ii)** Dose–response curve showing the IC₅₀ of compound D9 (24807456), with an IC₅₀ of 14.39 ± 2.4 µM obtained under identical conditions. Each data point represents the mean ± SD of three independent experiments (*n* = 3), each performed in technical triplicate. (**B**) Lineweaver–Burk plots illustrating the mode of inhibition by compounds D6 and D9. (**B**) **(i)** D6 (53747349) exhibits competitive inhibition with a K_i_ of 29 ± 10.2 µM, as seen by the convergence of lines on the y-axis with increasing inhibitor concentrations (0, 10, and 50 μM), indicating unchanged Vmax and increased K_m_. (**B**) **(ii)** D9 (24807456) exhibits competitive inhibition with a K_i_ of 7.2 ± 4.0 µM, as seen by the convergence of lines on the y-axis with increasing inhibitor concentrations (0, 10, and 50 μM), indicating unchanged Vmax and increased K_m_. Each data point represents the mean ± SD of three independent experiments (*n* = 3), each performed in technical triplicate.

Compounds that demonstrated substantial inhibition of PtpB-Mtb activity in the initial screening were further characterized to assess their potency and mode of inhibition. Dose–response line plots were generated for compounds 53747349 (D6) and 24807456 (D9) (Fig. 11A[i] and Fig. 11A[ii]), enabling determination of their half-maximal inhibitory concentrations (IC₅₀). Compound 53747349 (D6) inhibited PtpB-Mtb with an IC₅₀ value of 32.62 µM, while compound 24807456 (D9) exhibited a more potent inhibitory effect, with an IC₅₀ of 14.39 µM. The concentration-dependent inhibition observed for both compounds, as reflected in the characteristic sigmoidal curves, underscores their potential as effective enzyme inhibitors. To elucidate the inhibition mechanism, Lineweaver–Burk plots were constructed for both compounds by evaluating enzyme activity at varying concentrations of the inhibitors (0, 10, and 50 µM) (Fig. 11B[i] and Fig. 11B[ii]). In both cases, the lines corresponding to different inhibitor concentrations intersected at the y-axis, indicative of a competitive inhibition mechanism. This pattern was characterized by an increase in the apparent Michaelis constant (K_m_) without affecting the maximum velocity (V_max_), suggesting that the inhibitors compete directly with the substrate for binding at the enzyme’s active site. Collectively, these findings establish that the lead pyrazolo[4,3-c]pyridine/diazepane derivatives exhibit low-micromolar potency against PtpB-Mtb and function via a competitive inhibition mechanism, reinforcing their promise as targeted inhibitors of this virulence-associated phosphatase.

### Binding kinetics of hit compounds with PtpB-Mtb assessed by biolayer interferometry

**Fig 12:**
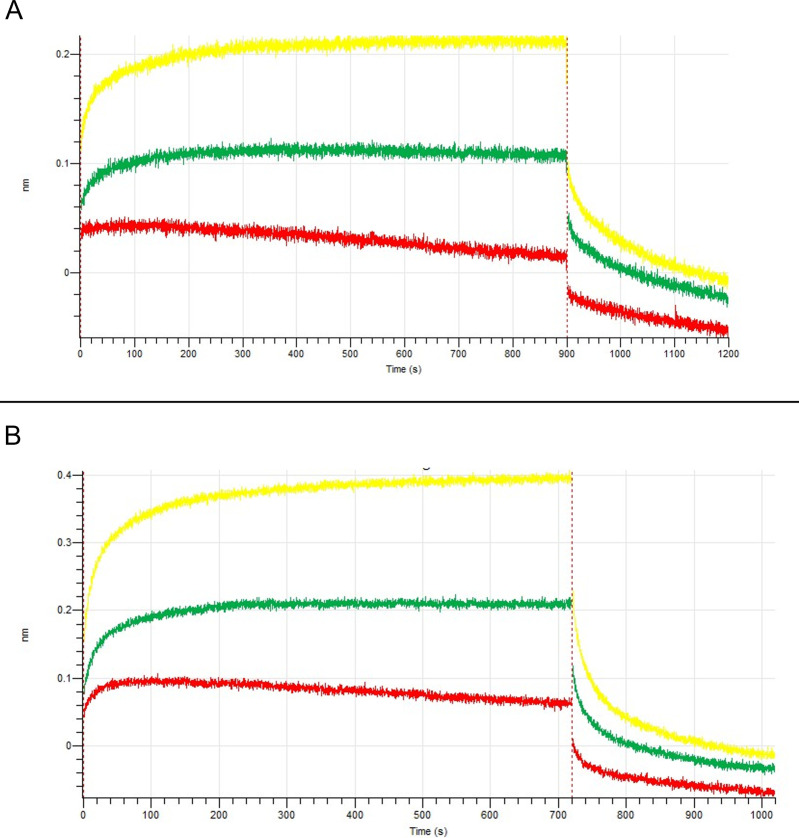
Binding analysis of compounds 53747349 (D6) and 24807456 (D9) with Mtb-PtpB using BLI. (**A**) and (**B**) show the BLI response plots depicting the binding kinetics of compounds 53747349 (D6) and 24807456 (D9), respectively, at varying concentrations to Mtb-PtpB (12.5 µM, 25 µM, and 50 µM) immobilized on Ni-NTA biosensors. Sensors with Mtb-PtpB but without compound (buffer only) were used as references, and the reference data were subtracted prior to fitting analysis. Data were fitted using the local binding model in the Octet BLI Systems Software. Dotted lines represent indicated raw data. The kinetic analysis revealed a dissociation constant Kd of 0.5704 µM for D6 and 0.0124 µM for D9, with a corresponding *R*² value of 0.94 for D6 and 0.96 for D9, indicating a strong and specific binding interaction with the target protein.

The binding interactions between PtpB-Mtb and the hit compounds 53747349 (D6) and 24807456 (D9) were assessed quantitatively using biolayer interferometry (BLI). Sensorgrams for each compound were recorded at three concentrations (12.5, 25, and 50 µM), with reference subtraction implemented to correct for non-specific binding and baseline drift. For compound 53747349 (D6) (Fig. 12A), a clear concentration-dependent increase in response was observed, indicative of a strong affinity toward immobilized PtpB-*Mtb*. Kinetic analysis revealed a dissociation constant (K_d_) of 0.5704 µM, with a coefficient of determination (*R*²) of 0.94, signifying a specific and stable interaction with the target enzyme. The relatively low K_d_ value denotes a substantial binding affinity, corroborating the compound’s potential as a viable inhibitor of PtpB-*Mtb*. Similarly, compound 24807456 (D9) (Fig. 12B) demonstrated robust, concentration-dependent binding, yielding a K_d_ value of 0.0124 µM and an *R*² of 0.96. The significantly lower K_d_ compared to compound D6 indicates an even greater binding affinity, which is particularly advantageous for the development of high-potency enzyme inhibitors. The pronounced binding strength of compound D9 underscores its potential as a lead scaffold for further optimization. Collectively, these kinetic parameters substantiate the capacity of both compounds to engage PtpB-*Mtb* with high affinity and specificity, with D9 displaying superior binding characteristics. These results complement the earlier docking and enzyme inhibition assays, providing a comprehensive biophysical validation of the lead molecules.

### Cytotoxicity assessment of hit compounds in HEK293T cells

**Fig 13:**
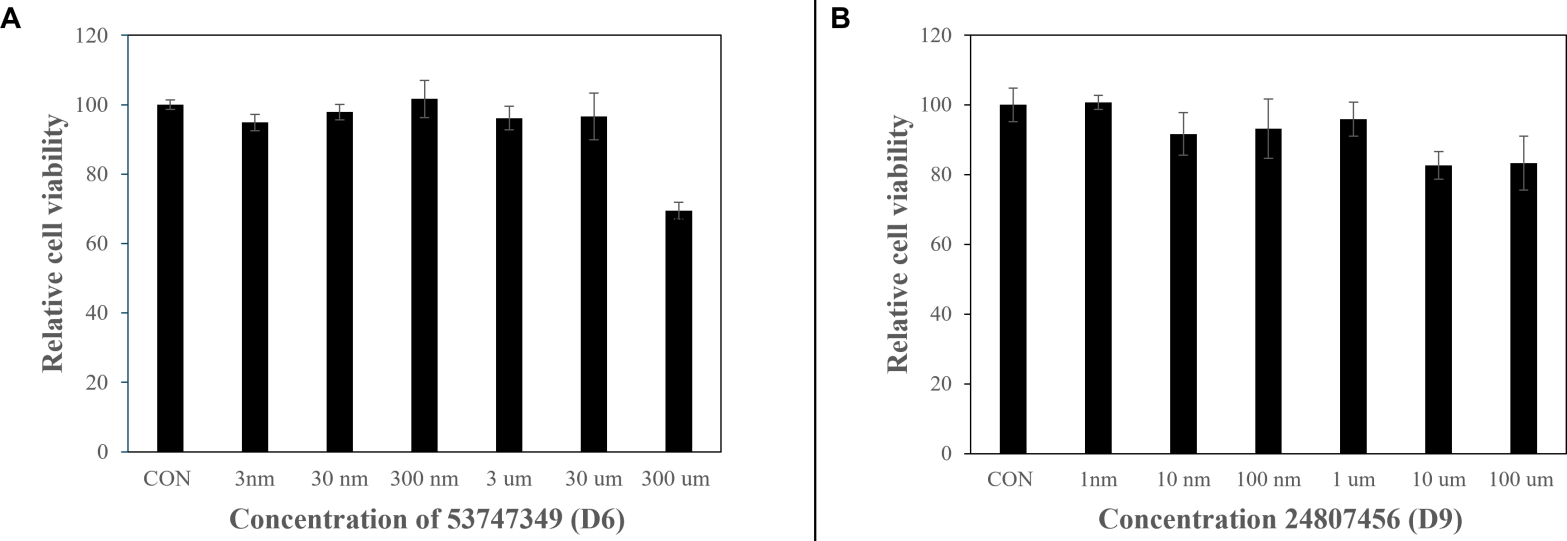
Cytotoxic effects of compounds 53747349 (D6) and 24807456 (D9) on HEK293T cells. HEK293T cells were seeded at a density of 5 × 10³ cells per well in 96-well plates and allowed to reach approximately 80% confluency. Cells were then treated with increasing concentrations of compounds (**A**) D6 (53747349) and (**B**) D9 (24807456) for 72 h. Cytotoxicity was assessed using the MTT assay, which measures mitochondrial metabolic activity as an indicator of cell viability. Following treatment, MTT reagent was added for 2 h to allow for the formation of insoluble formazan crystals, which were subsequently solubilized in DMSO. Absorbance was measured at 530 nm using a microplate reader. Relative cell viability was calculated as a percentage of the untreated control (CON). A significant reduction in cell viability was observed at the highest concentration of D6 (300 µM), while D9 showed minimal cytotoxicity across the tested concentrations.

The cytotoxic profiles of compounds D6 and D9 (corresponding to the hit candidates, 24807456 [D9] and 53747349 [D6]) were evaluated in HEK293T cells using the MTT assay across a broad concentration range. As depicted in Fig. 13, both compounds exhibited concentration-dependent effects on cell viability. For compound 53747349 (D6), cell viability remained above 90% at concentrations up to 30 µM, with a reduction observed at 300 µM (Fig. 13A). A pronounced decrease in viability that fell below the 70% cytotoxicity threshold was detected exclusively at the highest tested concentration of 300 µM (indicated by an asterisk), suggesting low cytotoxicity at pharmacologically relevant doses. Compound 24807456 (D9) displayed a similar trend, with relative cell viability consistently exceeding 85% across all concentrations tested (1  nM to 100 µM) (Fig. 13B). Even at the highest concentration (100 µM), viability remained above the cytotoxic threshold, indicating a favorable safety profile in HEK293T cells. These results demonstrate that both lead compounds maintain high cell viability over a wide range of concentrations, with only compound 53747349 (D6) exhibiting significant cytotoxicity at supra-physiological levels. This favorable cytotoxicity profile supports the continued investigation of these pyrazolo[4,3-c]pyridine/diazepane derivatives as potential PtpB-Mtb inhibitors. Collectively, these BLI kinetic analyses confirm that both compounds 53747349 (D6) and 24807456 (D9) bind directly and specifically to PtpB-*Mtb*, with measurable affinities in the low micromolar range.

## DISCUSSION

PtpB from *Mtb* is an established virulence factor implicated in immune evasion and intracellular survival (1–3). PtpB-*Mtb* functions as a secreted protein that modulates host immune responses, enabling the pathogen to persist and proliferate within macrophages. Genetic studies have demonstrated that deletion of the *ptpB* significantly impairs the bacterium’s ability to survive in guinea pig models and within macrophages, often resulting in lethal attenuation of the pathogen. This essentiality positions PtpB-*Mtb* as a scientifically validated and attractive target for the development of new anti-tubercular agents that disrupt *Mtb* survival mechanisms.

The identification of compounds D6 and D9 addresses several key limitations observed with existing PtpB inhibitor classes. Unlike the highly potent but membrane-impermeable oxamic acids (IC₅₀ ~6.4 nM), our compounds achieve a better balance of potency and drug-likeness. While D6 (IC₅₀ = 32.6 µM) and D9 (IC₅₀ = 14.4 µM) exhibit higher IC₅₀ values than the most potent reported inhibitors, they offer significant advantages in terms of predicted membrane permeability (TPSA = 88.3 and 67.6 Å², respectively, vs >120 Å² for oxamic acids), reduced molecular complexity, and favorable ADMET profiles. The inhibitory potencies of compounds D9 (14.39 μM) and D6 (32.62 μM) are modest when compared to the most potent PtpB inhibitors reported, including oxamic acid derivatives (IC₅₀ ~6.4 nM) and salicylic acid analogs (IC₅₀ ~38 nM). However, these values are characteristic of initial hits from screening campaigns and provide appropriate starting points for optimization. The tight binding affinity observed for D9 (Kd = 0.012 µM) suggests substantial potential for potency enhancement through rational design. Comparative analysis with recent computational drug design studies against *Mtb* further underscores the novelty of our approach. These studies primarily explored compounds targeting resuscitation-promoting factor B (RpfB) through virtual screening and molecular docking workflows (47, 48). In contrast, the present work focuses on PtpB, a secreted virulence phosphatase essential for immune modulation, thereby representing a distinct mechanistic target in Mtb pathogenesis. Moreover, the pyrazolo[4,3-c]pyridine and diazepane scaffolds identified here differ from the previously reported heterocyclic frameworks, highlighting both structural and mechanistic originality within the broader context of anti-TB drug discovery.

Notably, compound D9 demonstrated exceptional binding affinity (Kd = 0.012 µM) in BLI studies, which is superior to most reported PtpB inhibitors and suggests that kinetic binding parameters may be more predictive of biological activity than steady-state IC₅₀ values alone (49). This tight binding, combined with competitive inhibition kinetics, indicates effective active site occupancy that could translate to improved cellular potency (50).

The structural novelty of our scaffolds offers distinct advantages for medicinal chemistry optimization. Unlike the constrained oxamic acid or salicylic acid frameworks, the pyrazolo[4,3-c]pyridine and diazepane cores provide multiple sites for substitution and structural modification (51–53). The pyrazolo[4,3-c]pyridine scaffold (D6) incorporates a bicyclic heteroaromatic system that enables π–π stacking interactions while maintaining hydrogen bonding capability through the carboxylic acid moiety (53). The diazepane ring (D9) provides conformational flexibility and reduced planarity compared to aromatic inhibitors, potentially offering improved selectivity through shape complementarity rather than purely electrostatic interactions (54).

From a selectivity perspective, our compounds represent a departure from the phosphomimetic design strategy that has dominated PtpB inhibitor development (55). While compounds like OMTS rely heavily on mimicking the phosphotyrosine substrate through multiple negative charges, our scaffolds achieve binding through a combination of hydrogen bonding, hydrophobic interactions, and shape complementarity. This mechanism may provide enhanced selectivity against human PTPs, though direct selectivity profiling will be required to confirm this hypothesis (56).

In this study, a knowledge-driven virtual screening pipeline was implemented to identify novel chemotypes targeting PtpB-Mtb. Although the screening was not scaffold-restricted, two structurally distinct compounds—compound D6 (53747349) and compound D9 (24807456)—emerged as top hits. Retrospective structural analysis revealed the presence of pharmacologically privileged moieties: a pyrazolo[4,3-c]pyridine ring system in D6 and a 1,4-diazepane core in D9. Both scaffolds have garnered interest in medicinal chemistry due to their versatile bioactivity and drug-like properties, yet they remain underexplored in the context of mycobacterial pathogenesis, particularly PtpB inhibition.

Pyrazolo[4,3-c]pyridine represents a well-characterized heterocyclic framework extensively studied for its anticancer potential. Derivatives of this scaffold have been reported to inhibit various kinases—such as CK1, CHK1, PDK1, GSK-3, and PIM kinases—via both ATP-competitive and allosteric mechanisms (57–59). These compounds exert antiproliferative effects across several cancer cell lines by modulating p53 stability, inducing mitochondrial dysfunction, and activating caspase-mediated apoptosis (57). Despite this demonstrated pharmacological relevance, their application against Mtb proteins remains largely elusive.

In contrast, the diazepane ring system, although less characterized in the context of antimycobacterial therapy, is frequently found in CNS-active agents, kinase modulators, and protease inhibitors. Its seven-membered saturated ring imparts conformational adaptability and metabolic stability, traits considered favorable for enhancing drug–receptor interactions and pharmacokinetic performance (60, 61). While no direct precedent exists for diazepane-based inhibitors of PtpB, their structural and physicochemical advantages warrant further exploration in anti-TB drug discovery.

Compounds D6 and D9, incorporating these underrepresented scaffolds, exhibited promising inhibitory activity against PtpB-Mtb *in silico*. Their favorable docking scores and MMGBSA binding energies, combined with unique scaffold architectures and target-specific interactions, support their candidacy for further validation. Additionally, both molecules adhered to key drug-likeness parameters—including acceptable molecular weights, lipophilicity, hydrogen bonding potential, and TPSA—suggesting potential for oral bioavailability and *in vivo* tractability. The incorporation of multiple polar functional groups, akin to existing anti-TB drugs, may further enhance aqueous solubility and bio-distribution in host microenvironments.

MD simulations revealed that compounds D6 and D9 formed stable interactions with critical PtpB residues (Asp165, Lys166, Glu62) throughout the 500 ns trajectory, consistent with the robust hydrogen-bonding and hydrophobic interactions predicted by docking studies. Quantum chemical descriptor analyses further highlighted the favorable electronic profiles of both compounds, with narrow HOMO–LUMO gaps and negative free energies, indicative of chemical stability and reactivity suitable for protein interactions. The slightly lower ALIE value observed for D6 suggests a marginally higher electron reactivity, potentially contributing to its binding efficiency.

Interestingly, our selected compounds displayed a unique mode of interaction with PtpB-*Mtb*, characterized by strong electrostatic interactions and charge transfer mechanisms, diverging from the primarily hydrogen-bonding interactions reported for other PtpB inhibitors. This observation suggests potential for enhanced binding affinity and selectivity, offering opportunities for the rational design of next-generation inhibitors.

Biochemically, both compounds demonstrated low-micromolar potency in the pNPP phosphatase assay, with IC₅₀ values of 32.62 μM (D6) and 14.39 μM (D9). Although these values are higher than those reported for oxamic acid or salicylic acid, they represent a promising starting point considering the simpler synthetic routes, improved physicochemical properties, and better ADMET profiles afforded by the pyrazolo[4,3-c]pyridine/diazepane scaffold. Furthermore, Lineweaver–Burk analyses confirmed that both compounds act as competitive inhibitors, consistent with predicted active site binding and the classical substrate mimicry mechanism of PtpB inhibition (10).

BLI experiments revealed strong binding affinities, with K_d_ values of 0.5704 μM for D6 and 0.0124 μM for D9, the latter surpassing many reported small-molecule PtpB inhibitors in affinity. These kinetic data further reinforce the potential of these compounds as lead candidates for future medicinal chemistry optimization. Cytotoxicity assays in HEK293T cells demonstrated favorable safety profiles for both compounds, with only D6 exhibiting cytotoxicity at supra-physiological concentrations (>300  µM), suggesting an acceptable therapeutic window.

Despite these encouraging findings, several limitations remain to be addressed. This study focused exclusively on *in vitro* enzyme assays and computational analyses. While the compounds demonstrate biochemical inhibition of recombinant PtpB-Mtb and low cytotoxicity in mammalian cells, their efficacy in the natural intracellular context remains unvalidated. Given that PtpB exerts its virulence effects within infected macrophages where it modulates host immune responses and promotes mycobacterial survival, evaluation in macrophage infection models is essential. This cellular validation represents a critical next step for establishing the therapeutic potential of these scaffolds. Moreover, while the IC₅₀ values are promising, further optimization will be required to improve potency, selectivity, and pharmacokinetic properties. Structure–activity relationship (SAR) studies are planned to improve potency while maintaining favorable drug-like properties. Optimization strategies will focus on enhancing interactions with key catalytic residues (Asp165, Lys166, Glu62) identified through MD simulations. The pyrazolo[4,3-c]pyridine and diazepane scaffolds offer multiple derivatization sites for systematic modification, and their synthetic accessibility facilitates parallel synthesis approaches for comprehensive SAR exploration.

Building on these insights, future optimization efforts will involve selective modification of substituents around the pyrazolo[4,3-c]pyridine and diazepane cores to enhance permeability, selectivity, and inhibitory potency. Fine-tuning the electronic environment of the pyrazolo ring, through judicious incorporation of electron-donating or -withdrawing groups, may help balance lipophilicity and improve membrane penetration, while targeted substitution at the diazepane nitrogen could reinforce key active-site interactions. Such structure-guided refinements are expected to achieve an optimal balance between molecular reactivity and pharmacokinetic behavior, advancing these scaffolds toward more potent and selective PtpB inhibitors. Also, there is an absence of experimental selectivity validation against human phosphatases. While PtpB-Mtb exhibits low sequence homology to human PTPs and computational analyses suggest potential for selective interactions, these predictions require experimental confirmation through direct inhibition assays against PTP1B, TCPTP, SHP-1, SHP-2, and other relevant human phosphatases.

In conclusion, this work highlights the historical versatility and evolving potential of pyrazolo[4,3-c]pyridine and 1,4-diazepane as promising scaffolds for anti-tubercular drug development. Our identification of pyrazolo[4,3-c]pyridine and 1,4-diazepane derivatives as competitive PtpB-*Mtb* inhibitors, coupled with their favorable binding profiles, physicochemical properties, and low cytotoxicity, underscores their potential as attractive starting points for future optimization. Further efforts will focus on enhancing these leads through medicinal chemistry, validating their efficacy in cellular infection models, and advancing them toward preclinical evaluation.

### Conclusion

The present investigation has successfully identified and characterized novel pyrazolo[4,3-c]pyridine and 1,4-diazepane derivatives as potent inhibitors of PtpB-*Mtb*, a validated drug target in *Mtb*. Through a rigorous structure-based virtual screening strategy, coupled with MD simulations and QM analyses, compounds exhibiting high affinity, selectivity, and favorable drug-like properties were prioritized. Recombinant protein production and enzymatic assays confirmed the inhibitory activity of selected candidates, with several compounds demonstrating low micromolar IC₅₀ values against PtpB-*Mtb*. The integration of computational and experimental methodologies addressed key challenges of selectivity and cell permeability, underscoring the utility of this approach in anti-tubercular drug discovery. These findings provide a strong foundation for further optimization and preclinical evaluation of pyrazolo[4,3-c]pyridine/diazepane derivative -based PtpB-*Mtb* inhibitors, advancing the search for effective therapeutic interventions against drug-resistant TB.
